# The source and thermal driver of young (<3.0 Ga) lunar volcanism

**DOI:** 10.1126/sciadv.adv9085

**Published:** 2025-08-22

**Authors:** Chengyuan Wang, Yuqi Qian, Jintuan Wang, Liang Liu, Le Zhang, Zhiming Chen, Jingyou Chen, Guanhong Zhu, Xianglin Tu, Zexian Cui, Qing Yang, Yan-Qiang Zhang, Pengli He, Yonghua Cao, Haiyang Xian, James W. Head, Yi-Gang Xu

**Affiliations:** ^1^ State Key Laboratory of Deep Earth Processes and Resources, Guangzhou Institute of Geochemistry, Chinese Academy of Sciences, Guangzhou 510640, China.; ^2^ Department of Earth Sciences, University of Hong Kong, Hong Kong 999077, China.; ^3^ GIGCAS-HKU Joint Laboratory of Chemical Geodynamics, Guangzhou 510640, China.; ^4^ College of Earth and Planetary Sciences, University of Chinese Academy of Sciences, Beijing 100049, China.; ^5^ Department of Earth, Environmental and Planetary Sciences, Brown University, Providence, RI 02912, USA.

## Abstract

The thermal mechanism that drives the prolonged volcanism on the Moon, especially after the major pulse of Imbrian-aged eruptions, remains unknown. Here, we present a petrological and geochemical study of two types of young farside mare basalts, the ~2.8–billion year (Ga) low-Ti and ~2.9-Ga very-low-Ti basalts, collected during the Chang’e-6 mission. The results of our study show that these basalts have pyroxenitic sources and originate from a depth of ~60 to 80 kilometers and ~120 kilometers, respectively. The depth of their source that became progressively shallower over time, combined with thermal modeling results, suggests that magmatic underplating beneath the ilmenite-bearing cumulate (IBC) that escaped the mantle overturn phase could be a thermal driver for the young lunar magmatism. Global remote-sensing investigations further reveal different TiO_2_ contents between young basalts from each side of the Moon, attributable to asymmetric composition and thickness of the IBC in the uppermost mantle.

## Teaser

Melting of pyroxenite in the Moon’s uppermost mantle, driven by heat from magma underplating, has caused young lunar volcanism.

## INTRODUCTION

Over the decades, studies of lunar samples returned by the Apollo and Luna missions have demonstrated that mare volcanism occurred primarily between 3.9 and 3.1 billion years (Ga) (*1*–*3*). However, global crater size-frequency distribution measurements indicate the occurrence of younger mare volcanism during the Eratosthenian period (*4*–*6*). This observation is further confirmed by the recently returned ~2.0-Ga Chang’e-5 (CE-5) basalts (*7*, *8*). Young mare basalts are primarily found within the Procellarum KREEP Terrane, indicating a genetic relationship with KREEP material (rich in heat-producing elements such as K, Th, and U) (*5*, *9*). However, the CE-5 basalts contain negligible KREEP components (*10*), leaving the heat source for young volcanism an unsolved puzzle. Different from the deep peridotitic sources of the early Imbrian-aged Apollo mission-returned basalts (*11*–*13*), CE-5 basalts are thought to originate from a shallow pyroxenitic source (*14*–*16*). It remains unclear whether this is valid globally for young mare basalts or is specific to nearside CE-5 basalts.

**Fig. 1.**
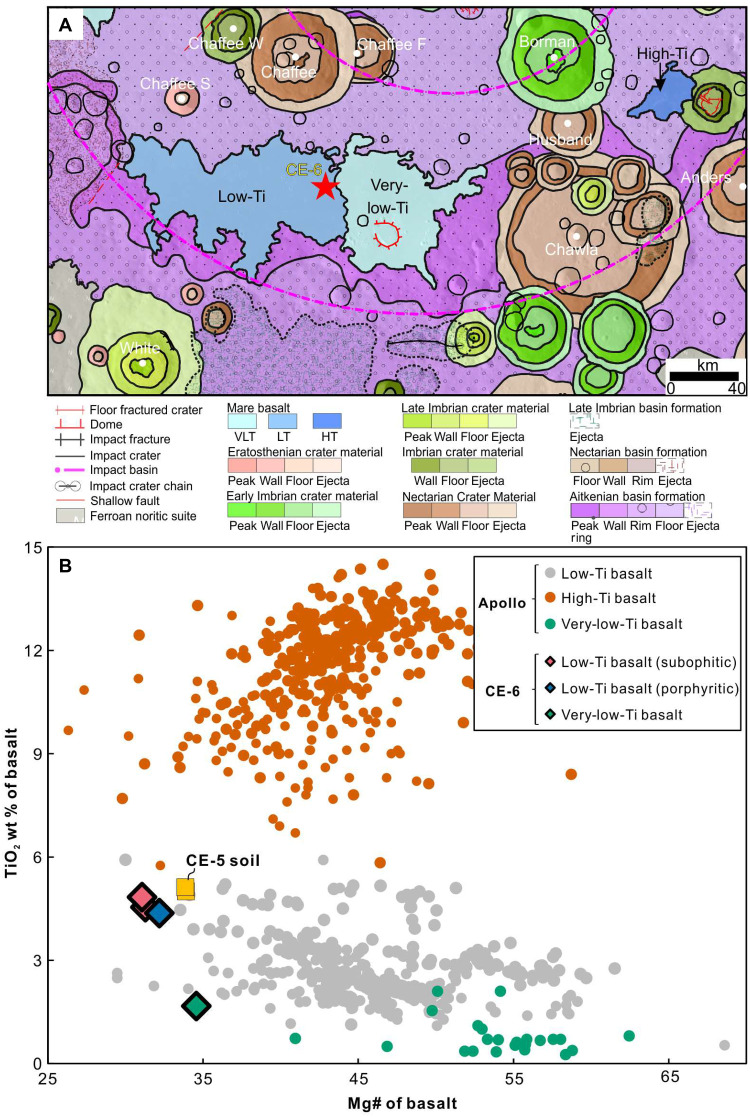
Geological context and compositions of the CE-6 LT and VLT basalts. (**A**) Location of the CE-6 sampling site (red star) in the southern part of the Apollo basin. The geological map is based on (*74*). (**B**) Mg# versus TiO_2_ in CE-6 low-Ti (LT) and very-low-Ti (VLT) basalts. CE-5 soil (*75*, *76*) and Apollo mare basalts (ApolloBasalt DB_V2 database) (*77*) are shown for comparison.

It has been shown that young volcanism not only is exclusive to the lunar nearside but also is reported from the South Pole-Aitken (SPA) basin at the lunar farside (*6*, *17*). Notably, the Moon has a notable nearside-farside asymmetries in geomorphology, crustal thickness, and chemical composition (*18*, *19*). Therefore, it is important to investigate whether the mechanisms behind young volcanism on the farside and the nearside (i.e., CE-5 basalts) are similar or not. Most recently, China’s Chang’e-6 (CE-6) mission returned farside samples from young basaltic units in the southern part of Apollo basin (Fig. 1A), situated in the northeast SPA basin. Two types of basalts sampled during the CE-6 mission, a low-Ti (LT) basalt and a very-low-Ti (VLT) basalt (*20*), were analyzed in this study. The nearby basalts on the east of the CE-6 landing site with lower TiO_2_ content were also found in the returned samples (Fig. 1). These two types of basalts offer an unprecedented opportunity to elucidate the variation of source lithology and formation conditions of young volcanism on the lunar farside.

In this study, we provide geochemical analyses of the basaltic samples, along with petrological investigations and thermal modeling. Our aim is to constrain the composition of their source, define the conditions under which they formed, and decipher the thermal drivers behind these young basalts. Our results, combined with global remote-sensing data, enable an insight into the temporal and spatial evolution of the Moon’s thermal regime.

## RESULTS

Preliminary studies show that CE-6 returned soils contain local basalt and abundant nonmare components (*21*). Two types of basalts within the CE-6 scooped samples, LT (*n* = 33) and VLT (*n* = 4), were initially identified (fig. S1) (*20*). For the LT basalts, in situ Pb-Pb dating and Sr isotopic analyses suggest that the CE-6 LT basalts formed at ~2.8 Ga [2830 ± 5 (1σ) million years (Ma) (*20*); 2807 ± 3 (1σ) Ma (*22*); and 2823 ± 6 (1σ) Ma (*23*)], with depleted initial ^87^Sr/^86^Sr of 0.69922 ± 0.00013 (2 SD) (*20*). In the VLT basalts, only two baddeleyites were found and their Pb isotopes significantly deviate from the 2.83-Ga isochron line of LT basalts (fig. S2 and data S1). The corrected Pb isotopes of baddeleyites (considering the influence of terrestrial and lunar common Pb) delineate an age of 2936 ± 26 Ma (1σ) (table S2). This older age of VLT basalts (relative to LT basalt) is in accordance with the crater counting chronology of the southern Apollo basin (*17*). Seven analyses of clean plagioclases in the VLT basalts yielded initial ^87^Sr/^86^Sr ratios (0.69948 ± 0.00019; fig. S3 and table S1) systematically higher than those of the LT basalts, providing further evidence for their different derivation.

The CE-6 LT basalts contain more ilmenite (~5 vol %) than the VLT basalts (<1 vol %), while compositionally zoned clinopyroxene (Cpx) and plagioclase are present in both suites (fig. S4). Olivine (Fo_59_) is rare and only observed in one of the LT clasts. Two typical textures, porphyritic (coarse-grained Cpx and plagioclase phenocrysts in a fine-grained matrix) and subophitic (similar grain sizes for Cpx and plagioclase, including poikilitic and equigranular textures), are noted for LT basalts. All the VLT basalts exhibit a subophitic texture (fig. S4).

**Fig. 2.**
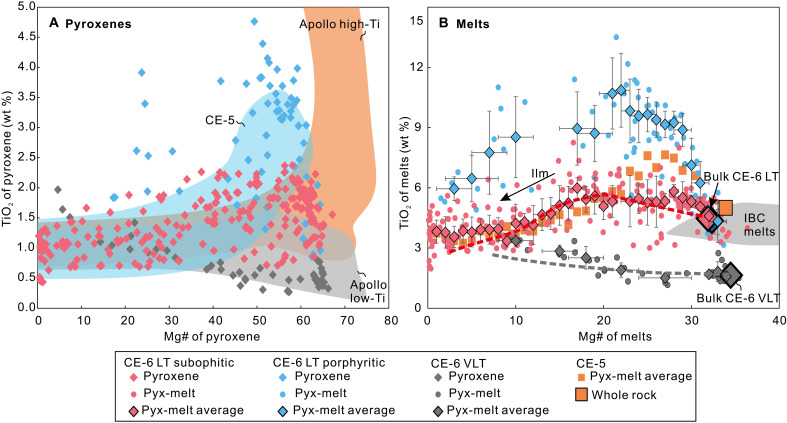
Pyroxene compositions and evolution of their parental melts of CE-6 basalts. (**A**) Mg# versus TiO_2_ in pyroxenes. The ranges of Apollo and CE-5 basalts are from (*16*). (**B**) Evolution trajectories of CE-6 LT and VLT basalts. Mg# and TiO_2_ in the equilibrated melts (pyx-melt) were calculated on the basis of pyroxene compositions (Supplementary Text). The vertical error bars are 1 SD for the average (pyx-melt average). The dashed lines display the evolution trajectories simulated by the PETROLOG program (*25*) using the bulk compositions of CE-6 LT and VLT basalts. Data of CE-5 basalts and the ilmenite-bearing cumulate (IBC) melts are from (*16*).

Pyroxenes from the CE-6 basalts contain lower abundances of the enstatite and diopside component, compared to Apollo LT and high-Ti (HT) basalts (fig. S1 and data S2), and display complex, texture-dependent compositional variations. While most magnesian pyroxenes in the porphyritic and subophitic CE-6 LT basalts are compositionally similar, the less enstatitic pyroxenes in porphyritic LT basalts have higher TiO_2_ content than those in subophitic LT basalts (Fig. 2A). Specifically, pyroxenes in the porphyritic LT basalts show a rapid increase in TiO_2_ content from ~1.5 to ~4.5 wt % when Mg# [defined as 100*Mg/(Mg + Fe)] decreases from ~64 to 50. Pyroxenes in the subophitic samples have TiO_2_ from 1 to 2 wt % over the same Mg# interval, followed by a gradual decrease to ~1 wt % with decreasing Mg# (Fig. 2A). The pyroxenes from VLT basalts differ significantly from those in the LT suite, with TiO_2_ < 0.5 wt % and an overall increase in TiO_2_ as Mg# decreases (Fig. 2A).

**Fig. 3.**
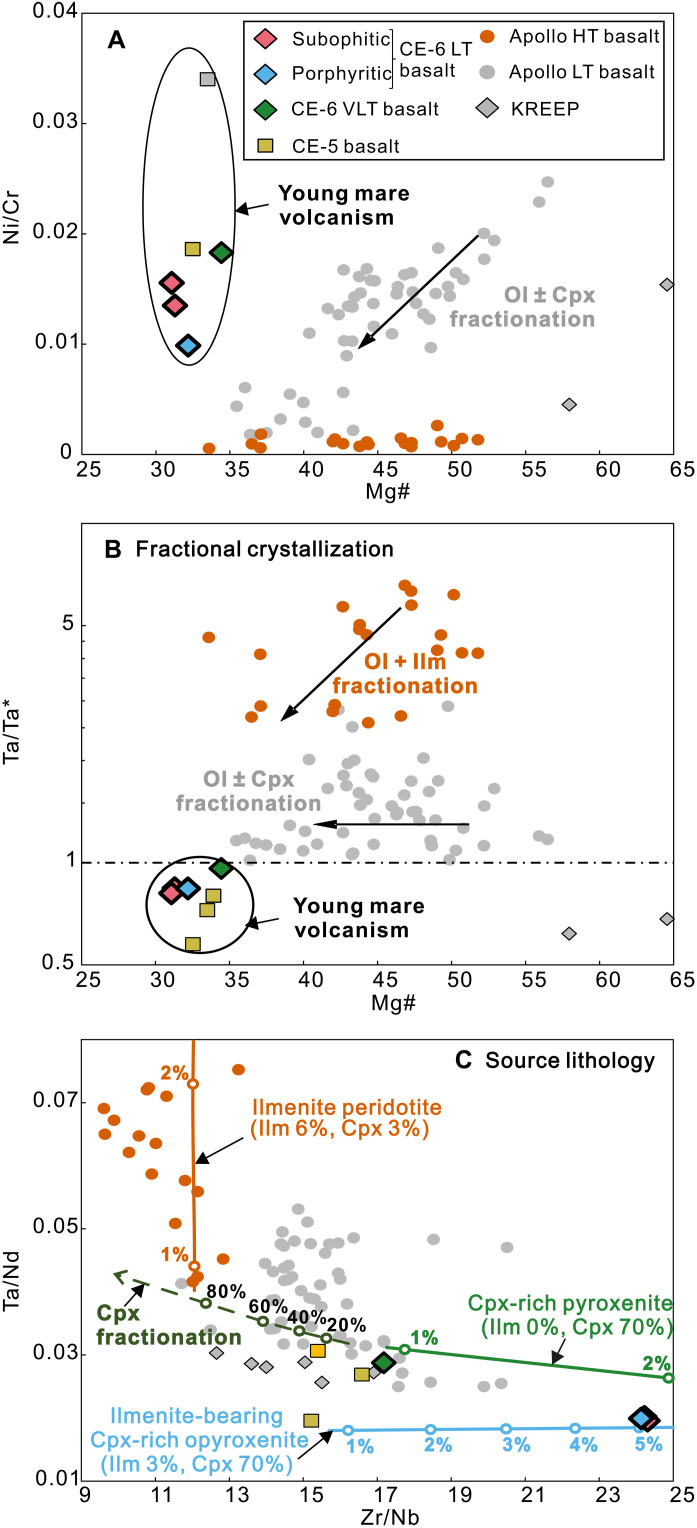
Geochemical variability of mare basalts and modeled trends of fractional crystallization and nonmodal mantle melting. (**A**) Ni/Cr versus Mg#; (**B**) Ta/Ta* versus Mg# of bulk rocks. Data of the Apollo basalts (solution ICP-MS data) (*12*, *78*), the KREEP materials (*12*, *30*, *78*), and CE-5 basalts (*16*, *76*, *79*) are also shown for comparison. (**C**) Ta/Nd and Zr/Nb in melts generated by melting of different mantle sources. The solid curves display trends of nonmodal partial melting of ilmenite peridotite (6% Ilm and 3% Cpx), Cpx-rich pyroxenite with no ilmenite (~70% Cpx), and ilmenite-bearing Cpx-rich pyroxenite (~3% Ilm and ~70% Cpx), respectively. The source compositions are calculated from (*30*). Numbers on the curves denote melting degree. The dashed curve denotes the fractionation trend of clinopyroxene (Cpx) in mare basalts using Apollo LT (12064) as a parental magma. Numbers on the curve denote proportion of fractionation. See Supplementary Text for the details of modeling.

Two subophitic LT clasts (S3551-P1, 3.5 mm; and aS3576-P1, 2.5 mm), one porphyritic LT clast (S3576-P3, 2.5 mm), and one VLT clast (S3576-P4, 1.5 mm) were digested for bulk major and trace element analysis (see Materials and Methods). The three LT basaltic clasts yield consistent bulk compositions (tables S3 and S4). Compositionally, they are classified as LT (4.38 to 4.83 wt %)/low-Al/low-K mare basalts (*24*) with low Mg# (31 to 32) (Fig. 1B), similar to the CE-5 basalts (TiO_2_ = 5 wt %, Mg# = 33.9) (table S3). The CE-6 VLT basalt has marginally higher TiO_2_ (1.67 wt %) content and lower Mg# (34.6) than the Apollo VLT basalts (Fig. 1B). It is, nevertheless, classified as VLT basalt according to their pyroxene compositions (fig. S1). The LT basalts exhibit similar trace element patterns to the VLT basalts, but with higher concentrations (fig. S5). Both types display elevated Ni/Cr, negative Ta anomaly [Ta/Ta* = 2*Ta_N_/(Th_N_ + Ce_N_), N subscript means chondrite normalized], lower Ta/Nd, and higher Zr/Nb compared to the Apollo mare basalts (Fig. 3).

## DISCUSSION

### Constraints on the lithology of the source region

Pyroxenes crystallized in the basalts can record the compositional variation of the equilibrated melts. Here, we use the TiO_2_-Mg# data of Cpxs in the CE-6 basalts to calculate variation of TiO_2_-Mg# in the melts during basaltic evolution (Supplementary Text) (Fig. 2A). Results show that the most primitive parental melts are virtually identical (~4.3 wt % TiO_2_, Mg# = ~33) for both subophitic and porphyritic LT basalts, aligning with their bulk compositions (Fig. 2B). The reconstructed fractionation trajectory for the CE-6 LT basalts consists of two stages (Fig. 2B), in agreement with the simulation result by PETROLOG program (*25*) and similar to what has been suggested for the CE-5 basalts (*16*). The early stage is characterized by an increasing TiO_2_ with decreasing Mg# to ~20, corresponding to initial crystallization of Cpx and plagioclase as indicated by the petrography (fig. S4). Subsequent crystallization of ilmenite resulted in a decline in TiO_2_ in the residual melts. Specifically, TiO_2_ content of the porphyritic LT basalts increases more significantly in the earlier stage, indicating a higher degree of crystallization. This is in line with its porphyritic texture with coarse-grained Cpx and plagioclase phenocrysts (fig. S4). The porphyritic texture implies that rapid cooling and ilmenite crystallization may have been suppressed during this process. This is supported by the petrographic observation that ilmenite is only found as small crystals within the matrix (fig. S4) and explains the higher TiO_2_ of pyroxenes in the porphyritic basalt than in the subophitic LT basalts. The CE-6 VLT basalts lack the ilmenite crystallization stage in comparison with the LT basalts. This is consistent with their petrography (fig. S4) and explains the trend of monotonically increasing TiO_2_ content with decreasing Mg# in Fig. 2B. The subophitic and porphyritic textures may originate from different parts within a basalt flow (with porphyritic sample cooling more slowly than the subophitic sample). These LT basalts likely underwent distinct evolutionary trajectories and lack a genetic relationship with the VLT basalts, as indicated by their different TiO_2_-Mg# correlation in Cpx and different initial ^87^Sr/^86^Sr ratios (Fig. 2 and fig. S3).

Mare basalts with low Mg# are commonly attributed to extensive fractionation of a magnesian parental magma (*9*–*11*). However, this process has been debated for CE-5 basalts (*16*, *26*) and cannot be reconciled with the trace element compositions of the CE-6 basalts, given the following considerations: (i) Extreme fractionation from an Apollo LT type basalt would result in an enrichment of incompatible elements (*10*), which is not observed in CE-6 basalts (fig. S5). (ii) The CE-6 basalts have higher Ni/Cr (0.01 to 0.02) at comparable Mg# and plot significantly away from the fractionation trends of high Mg# Apollo LT and HT types of basalts, arguing against a genetic link between them (Fig. 3A). (iii) The Apollo LT and HT basalts generally show higher Ta/Ta* [defined as 2*Ta_N_/(Th_N_ + Ce_N_), N subscript means chondrite normalized] and Ta/Nd than the CE-6 basalts (Fig. 3, B and C). Because Ta is more compatible than the rare earth elements in ilmenite (*27*), fractionation of ilmenite may partly explain the low Ta/Ta* and Ta/Nd in the CE-6 basalts. However, as illustrated above, ilmenite fractionation did not occur until the late evolution stage of CE-6 basalts (Fig. 2B). Addition of KREEP is also precluded given the depleted Sr-Nd isotopes in CE-6 basalts (fig. S3) (*20*). (iv) Cpx fractionation would lead to a low Zr/Nb ratio in the melt, as it has D_Zr_ ≫ D_Nb_ (*28*). Therefore, the higher Zr/Nb of the CE-6 basalts in comparison to the Apollo basalts cannot be explained by this process (Fig. 3C).

We therefore propose that the trace element features of the CE-6 basalts reflect the presence of Cpx and ilmenite in their source regions (*15*, *16*). Because the influence of fractional crystallization on Zr/Nb and Ta/Nd is excluded on the basis of the discussion above, we can then quantitatively estimate the mineral assemblage in the source by modeling the variation of these ratios in melts (Supplementary Text) using ilmenite-bearing peridotite and Cpx-rich pyroxenite as source materials. We applied a nonmodal melting model on the basis of the melting kinetics, which suggests that Cpx should have contributed more than ilmenite to the melts from the source (*29*). Ilmenite has D_Ta_/D_Nd_ ≫ 1, while the opposite is true for Cpx (D_Ta_/D_Nd_ < 1) (*27*, *30*). Consequently, ilmenite in the residue during nonmodal melting would scavenge Ta and lower Ta/Nd ratio in melts. In addition, because Cpx has a much higher Zr/Nb ratio than ilmenite (*30*), partial melting of a Cpx-rich pyroxenite can produce high Zr/Nb ratio in the CE-6 LT basalts (Fig. 3C). The modeling results show that a source with ~70 wt % Cpx and ~3 wt % ilmenite is capable of producing the high Zr/Nb and low Ta/Nd in the CE-6 LT basalts (Fig. 3C). Therefore, an ilmenite-bearing Cpx-rich pyroxenite source, with a lithology similar to the ilmenite-bearing cumulate (IBC), the late-stage cumulate of the lunar magma ocean (LMO) that is mainly composed of ~60 to 90 wt % Cpx and ~5 to 20 wt % ilmenite (*31*–*34*), is proposed as the source lithology of the CE-6 LT basalts. The source resembles a cumulate composition similar to the one suggested for CE-5 basalts on the lunar nearside (*15*, *16*). But compared to what has been suggested for CE-5 basalts (~10 to 15 wt %), the source of CE-6 LT basalts might contain less ilmenite (~3 wt %). As for the CE-6 VLT basalts, the source should also be Cpx-rich pyroxenite but contains negligible ilmenite. A source composition dominated by Cpx containing traces of ilmenite can further explain high Ni/Cr and Ta/Ta*: Cpx in the source can scavenge Cr from the melt [D_Cr_ ≫ 1; (*28*)], while olivine [D_Ni_ ≫ 1; (*35*)], which can hold Ni, is lacking in the pyroxenite source; ilmenite in the source can scavenge Ta from the melt, while Ce and Th prefer to partition into the melt [D_Ta_ > 1 ≫ D_Ce&Th_; (*27*)].

### A shallow IBC source modified by the SPA impact

**Fig. 4.**
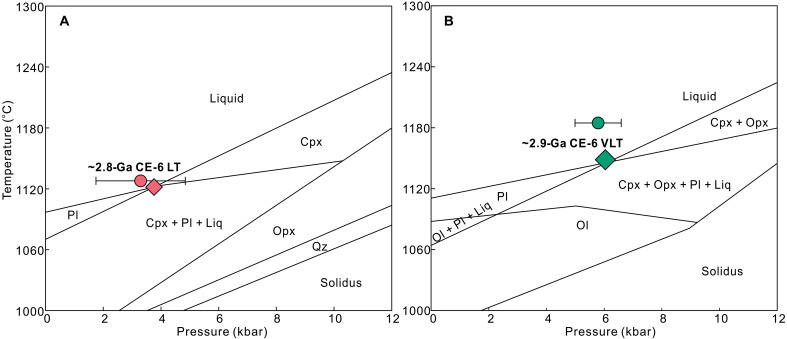
Phase equilibrium calculations of the CE-6 basalts. (**A**) shows LT and (**B**) shows VLT basalts. The diamond denotes the P-T condition of the multiple saturation point (MSP). The circle denotes the result from mineral thermobarometric calculation (see Discussion for details). Cpx, clinopyroxene; Ol, olivine; Pl, plagioclase; Opx, orthopyroxene; Qz, quartz; Liq, liquid.

On the basis of our observations and modeling results, we suggest that the CE-6 LT basalts represent primary magmas of an IBC source, rather than being products of extensive fractionation from a peridotite-derived melt: (i) As we discussed above, modeling of trace elements argues against fractionation process and requires an IBC source for the CE-6 LT basalts; (ii) partial melts of the IBC source are able to produce low-Mg# and intermediate TiO_2_ contents that are featured by the CE-6 LT basalts (Fig. 2B); (iii) the most magnesian pyroxenes are in equilibrium with the bulk compositions of the CE-6 LT basalts (Fig. 2B), while olivine is rarely observed. Accordingly, the multiple saturation point (MSP) of the CE-6 LT basalts is expected to be close to the pressure-temperature (P-T) condition, under which the primary magma was segregated from its source (*11*, *36*, *37*). Here, we use the MSP obtained by the program GeoPS (*38*) together with results of thermobarometers to constrain the formation conditions of basaltic melts. To evaluate the validity of these methods, we conducted piston-cylinder experiments at conditions between 0.4 and 1.3 GPa and between 1100° and 1230°C on starting materials with CE-5 basalt compositions. The experimentally determined MSPs were then checked against the conditions of experimental runs. As shown in fig. S6 and data S3, the MSP modeling results using the program GeoPS (*38*) match well with the experimental conditions on CE-5 and Apollo basalts. The Cpx-liquid barometer (*39*) and plagioclase-liquid thermometer (*40*) also provided relatively consistent pressure and temperature estimates (fig. S6). We then applied these approaches to the CE-6 basalts. The modeled phase relation yields a MSP for the CE-6 LT basalt at ~3 to 4 kbar and ~1120° to 1140°C, where Cpx and plagioclase are saturated (Fig. 4A), indicating the presence of plagioclase in the source. The lack of olivine in Fig. 4A matches with our petrographic observation (fig. S4) and supports a pyroxenite source, distinguishing them from those of the Imbrian-aged Apollo mare basalts (*37*). Similar results are obtained by the Cpx-liquid barometer (3.3 ± 1.6 kbar) and plagioclase-liquid thermometer (1128°C) (Fig. 4A). Thus, we suggest that CE-6 LT basalts are most likely to have originated from a shallow IBC source, similar to what has been suggested for CE-5 basalts (*14*–*16*). The estimated depth (~60 to 80 km) of this IBC source is in good agreement with the prediction by the LMO hypothesis (*34*, *41*), further implying that the overturn of IBC may be incomplete (*42*–*44*).

**Fig. 5.**
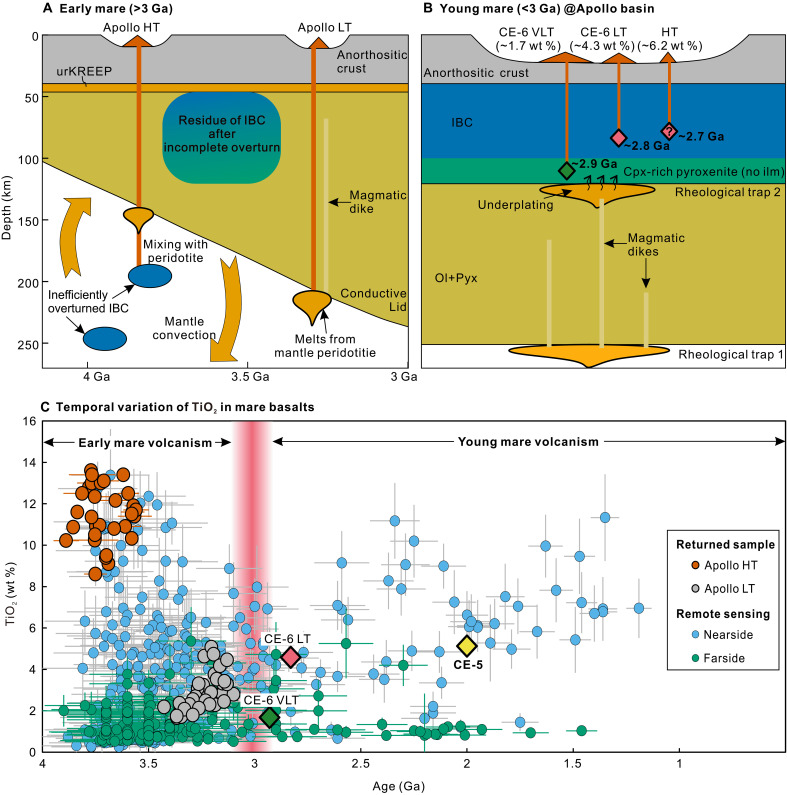
Distinct thermal regimes, variation of source depths, and secular change of TiO_2_ contents in old (>3.0 Ga) and young (<3.0 Ga) mare basalts. (**A**) Schematic diagram illustrating the potential thermal driver from mantle for early mare volcanism (>3 Ga). After LMO solidification, overturn was incomplete on a global scale with IBC partially sinking into the deep mantle. The inefficiently overturned IBC domain and mantle peridotite within convective mantle melted as the source of the Imbrian-aged HT and LT basalts collected by Apollo missions. Relatively thin lithosphere before 3 Ga allowed surface eruption of the melts from convective mantle. (**B**) The interpreted source and thermal driver for the CE-6 basalts in the Apollo basin. The remaining IBC is proposed to be present in the uppermost mantle beneath this area. The thickened lithosphere after 3 Ga inhibited surface eruption of melts from convective mantle and resulted in underplating of these melts underneath rheological traps. The orange drop-shaped patches denote the underplated melts. The HT basalt is located in the southeastern Apollo basin (Fig. 1A), with its age and TiO_2_ derived from remote-sensing data analysis (*17*). Mantle structure is based on (*16*, *34*, *41*). (**C**) Global Clementine remote-sensing data (data S4) (*61*) show the secular variations in TiO_2_ contents in mare basalts from ~4 to 1 Ga. Data of the Apollo HT and LT basalts are from (*1*). The emplacement ages of mare basalts are compiled from (*4*–*6*, *62*).

As suggested by previous geodynamic studies, the density disparity between IBC and the underlying peridotite may have led to a large-scale overturn of LMO (*45*, *46*). This process likely introduced chemical heterogeneities into the lunar mantle (Fig. 5A). As shown in Fig. 3C, the Zr/Nb and Ta/Nd variation of the Apollo HT basalts can be modeled by melting of an ilmenite peridotite source, which requires mixing of overturned IBC and the early-stage cumulate of LMO in the deep mantle, consistent with the experimental study and the Fe-Mg-Ca isotopes of HT basalts (*35*, *43*, *47*). On the other hand, recent geodynamic models argue for partial sink of IBC into the deep mantle (*42*) and incomplete overturn (*43*, *44*) (Fig. 5A). Our research on the CE-6 basalts supports the incomplete overturn hypothesis and suggests the remaining IBC at the uppermost mantle beneath the Apollo basin as their sources (Fig. 5B).

The bulk compositions of the relatively older CE-6 VLT basalts (~2.9 Ga) are in equilibrium with their primitive (early-formed) mineral phases (Fig. 2B). Therefore, the MSP of these basalts, along with mineral thermobarometric calculation results, can also provide constraints on the P-T conditions of their source region. The modeled MSP is at ~5 to 6 kbar and ~1140° to 1160°C, in good agreement with thermobarometric estimates (5.8 ± 1.6 kbar, 1185°C) (Fig. 4B). Clearly, the ~2.9-Ga CE-6 VLT basalts originated from a deeper Cpx-rich pyroxenite source (~100 to 120 km) than the ~2.8-Ga LT basalts (Fig. 5B). The saturation of Cpx, plagioclase, and orthopyroxene at MSP indicates the presence of more Ca-poor pyroxenes in the source (Fig. 4B), supporting their Cpx-rich pyroxenite source as relatively earlier stage cumulate before the formation of IBC during LMO solidification (*13*). Notably, spectral data indicate that the relatively younger (~2.7 Ga) HT basalt located eastward of CE-6 landing site (Fig. 1A) (~100 km from the VLT basalt) has FeO (~18 wt %) and TiO_2_ content (~6.2 wt %) similar to the ~2.8-Ga LT basalts (*17*), suggesting a similar derivation. Together, these observations indicate that the young volcanisms in the southern Apollo basin show source migration from the deep-seated Cpx-rich pyroxenite at ~100 to 120 km to the shallower IBC at ~60 to 80 km (Fig. 5B).

As the late-stage cumulate of LMO, IBC is expected to be enriched in incompatible elements (i.e., high Rb/Sr and low Sm/Nd ratios). However, Sr-Nd-Pb isotopes of the CE-6 LT basalts are highly depleted (*20*, *23*), especially when compared to those of the CE-5 basalts (*10*). Because they are both supposed to be derived from the IBC, a possible explanation is that the shallow mantle beneath SPA basin may have been modified by the SPA impact at ~4.25 to 4.33 Ga (*48*, *49*). This giant impact was supposed to have induced decompression melting of the underlying mantle with the melts being extracted into the impact melt sheet (*50*–*52*). Assuming the ^87^Rb/^86^Sr (0.017) and ^147^Sm/^144^Nd (0.232) of the CE-5 basalt source represent those of the unmodified IBC, we show that, after low degree (~2 to 5%) of melt extraction during the SPA impact, the modified IBC should have lower ^87^Rb/^86^Sr (0.005) and higher ^147^Sm/^144^Nd (0.29), which explains the Sr-Nd isotopes of the CE-6 LT basalts (fig. S7). Although the Nd isotopes of CE-6 VLT basalts were not measured because of the lack of Nd-rich phase (i.e., merrillite), their Sr isotopes are more enriched than the LT basalts (fig. S3), indicating a less modified mantle source (fig. S7). This is reasonable because the VLT basalts were derived from a deeper source than the LT basalts and, hence, may have experienced less melt extraction during the SPA impact. An alternative mechanism for the formation of the Cpx-rich pyroxenite source of CE-6 LT basalts is a cumulate of the SPA impact melt. However, so far, the proposed cumulate in the deep part of the SPA impact melt sheet is dunite and orthopyroxenite (*51*), rather than Cpx-rich pyroxenite. Besides, the SPA impact melt sheet is supposed to be 50 km in depth (*50*), shallower than the source of CE-6 LT basalts (~80 km) (fig. S7).

### Thermal driver and possible dichotomy of young mare basalts

Based on the discussions above, the source of young (<3 Ga) mare basalts is interpreted as Cpx-rich pyroxenite in the uppermost mantle that escaped the earlier mantle overturn. Such a source contrasts with the typical peridotite mantle source commonly accepted for old (>3 Ga) mare basalts represented by Apollo samples (*12*, *13*). The shallow IBC source for the young lunar volcanism represented by CE-5 and CE-6 basalts indicates that the heat source is potentially from tidal heating, impact-induced heating, subcrustal KREEP layer, and deep mantle beneath IBC. Here, we propose that the thermal driver lies below the IBC for the following considerations: (i) Tidal heating may have contributed to the magmatic activity of the early Moon, when the distance between the Earth and the moon was relatively close (<19 Earth radii) (*53*). If the young mare volcanism was driven by tidal heating, then basalts like CE-5 and CE-6 would likely be more prevalent in early basaltic volcanism. This is inconsistent with the lack of CE-5– and CE-6–type samples in the Apollo collection. (ii) Data from the CE-6 samples have demonstrated that the impact flux on both the lunar farside and nearside is similar, with both markedly decreased since the late heavy bombardment (*20*, *48*). Besides, notable Eratosthenian-aged impact craters are rare in the Apollo basin (*17*), making it challenging to induce melting of the IBC at ~100 km depth. (iii) Heat from a subcrustal KREEP layer would first cause melting of the IBC and then the underlying Cpx-rich pyroxenite, in contrast to our findings (Fig. 5B). Moreover, the KREEP signature is negligible in CE-5 and CE-6 basalts (*10*, *20*). (iv) Cooling of the Moon could be slow due to the outer megaregolith layer that may serve as a thermal lid (*54*). The persistence of lunar dynamo recorded by CE-5 and CE-6 basalts supports deep mantle convection until ~2 Ga (*55*, *56*), indicating sufficient heat sustained in the Moon’s interior for the young mare volcanism.

To understand how heat from the deep mantle supplied to the melting of shallow IBC, we propose a thermal model of underplating beneath the remaining IBC layer (fig. S8). Following the major phase of mare volcanism (~3.9 to 3.0 Ga), the thickened lithosphere inhibited surface eruption of melts from the convective mantle (*11*, *45*, *54*). These melts were likely to pool or trap when encountering a rheological trap (boundary between layers with different rheological properties) in the lithosphere, forming extensive sills beneath the upper layer (*57*, *58*). Basal melting of the upper layer would be driven by heat conductively transferred from large volumes of underplated melts. Here, we use a numerical model to simulate this process (Supplementary Text) (fig. S8). One notable rheological trap is at the lithospheric base (Fig. 5B; rheological trap 1); however, our numerical modeling shows that the shallow IBC cannot be melted even with very high underplating flux (~3 km/Ma) at this depth (fig. S8B). Besides, magmatic dikes can still rise from lithospheric base toward the surface and reach the remaining IBC in the uppermost mantle, as suggested by the recent GRAIL gravity model which reveals dikes of 1.2 × 10^4^ km^3^ within the lithosphere beneath the Apollo basin (*59*). Differences in porosity and rheological properties between the IBC (pyroxenite with low porosity) and underlying peridotite (*57*, *58*, *60*) are likely to inhibit percolation of magmatic dikes and cause underplating at the IBC base (Fig. 5B; rheological trap 2). Our numerical modeling calculations show that low flux (~0.2 km/Ma) and high flux (~2 km/Ma) of underplated melts at this depth can raise geotherms at ~110 km (Cpx-rich pyroxenite) and ~80 km (IBC) above the solidus, respectively (fig. S8, C and D). The migration of sources of the CE-6 basalts from ~110 to ~80 km may reflect an increase in underplating flux, likely occurring below SPA-impact basin, possibly due to thinning of the lithosphere through basin-induced effects.

To test whether this thermal mechanism can be applied to the whole Moon, we compiled global TiO_2_ abundance data obtained by Clementine (*61*) and model ages of mare basalts based on crater-counting method (*4*–*6*, *62*). We show that, after 3 Ga, the volcanism on the farside is dominated by VLT basalts (average TiO_2_ = 1.6 ± 1.2 wt %, *n* = 30), similar to the CE-6 VLT basalts (TiO_2_ = 1.67 wt %) (Fig. 5C). On the nearside, the young basalts (<3 Ga) exhibit relatively higher TiO_2_ contents (average = 5.4 ± 2.6 wt %, *n* = 66), similar to the CE-6 LT basalts and CE-5 basalts (TiO_2_ = ~4.6 to 5.1 wt %) (Fig. 5C). The difference in TiO_2_ contents between young basalts from each side of the Moon indicates that the lunar dichotomy also exists in the compositions of mare volcanism, which may reflect asymmetry of the lunar mantle (*4*, *22*). Here, we propose that this difference is likely caused by asymmetric composition and thickness of IBC in the uppermost mantle (fig. S9). On the basis of interpretation of sources for CE-5 and CE-6 basalts, the remaining IBC in the nearside contains ~10 to 15 wt % ilmenite at ~100-km depth (*14*, *16*), while the farside IBC only contains ~3 wt % ilmenite at ~60 to 80 km (Figs. 3 and 4). This difference indicates a thicker IBC with more ilmenite in the nearside’s uppermost mantle than that of the farside (fig. S9), probably as a result of heterogeneous distribution of IBC throughout the mantle (*42*) or the SPA impact (*46*, *52*). However, we suggest that the asymmetrical LMO crystallization is a likely cause (*63*): The earlier crystallized farside’s LMO would have produced less late-stage IBC. Consequently, after 3.0 Ga, melting of the nearside’s thick and ilmenite-rich IBC can produce CE-5 like basalts, whereas melting is largely confined to the basal Cpx-rich pyroxenite layer on the farside because of the thin and ilmenite-poor IBC layer (fig. S9). In the latter scenario, volcanism like CE-6 VLT basalts would occur on the farside rather than limited within the SPA basin (Fig. 5C).

Another implication is that the lunar thermal regime is not only spatially different (*64*) but may also have changed at ~3 Ga. Before ~3.0 Ga, multiple heat sources such as tidal heating, impacts, underplating beneath the lithosphere or shallow IBC, and the subcrustal KREEP, as well as diverse sources (peridotite + IBC) may all have contributed to Imbrian-aged volcanic activity (Fig. 5A). Afterward, young mare volcanism was primarily driven by magmatic underplating beneath the shallow IBC (Fig. 5B), as other heat sources diminished. This interpretation suggests that the IBC may also be the source of some Apollo samples, which can be tested by further analyses of these samples.

## MATERIALS AND METHODS

### SEM and EPMA analyses

The studied basaltic clasts collected from two aliquots of the CE-6 lunar soils (scooped sample CE6C0100YJFM003 and CE6C0200YJFM004) and reference materials were embedded in epoxy resin mounts and then polished with a fine (1 μm) diamond suspension using a grinder. The petrographic examination was carried out on a Zeiss Gemini 450 field emission scanning electron microscope (SEM) at the Guangzhou Institute of Geochemistry, Chinese Academy of Sciences (GIGCAS). The accelerating voltage was 15.0 kV, and the probe current was 2.0 nA. An energy-dispersive spectrometer was used to identify mineral phases including baddeleyite. The major element concentrations in the minerals (pyroxene and plagioclase) were analyzed by a JEOL JXA-8230 electron probe microanalyzer (EPMA) at the Key Laboratory of Mineralogy and Metallogeny, GIGCAS. Analyses were performed using an accelerating voltage of 15 kV, probe current of 20 nA, and electron beam diameter of 1 μm. Peak and background counting times were 10 and 5 s for K and Na, 20 and 10 s for major elements of Cpx (Si, Fe, Al, Mg, and Ca) and plagioclase (Si, Al, and Ca), and 40 and 20 s for other minor elements, respectively. Overlap correction was taken to subtract the interference of Ti on V. The standards for calibration are Cr-diopside (Si and Ca), magnetite (Fe), olivine (Mg), almandine (Al), rutile (Ti), rhodonite (Mn), orthoclase (K), albite (Na), V metal (V), apatite (P), and Cr2O3 (Cr). ZAF (where these letters stand for atomic number, absorption and fluorescence) calibration procedures were used for reducing matrix effects.

### Major and trace elemental analyses by ICP-MS

Bulk major and trace element concentrations of the four basaltic clasts were determined by inductively coupled plasma mass spectrometry (ICP-MS) at the GIGCAS. For each sample, a mass of 1.39 to 27.5 mg was weighed into a precleaned 7-ml perfluoroalkoxy (PFA) beaker and digested with a 2:1 (v:v) mixture of concentrated HF and 8 M HNO_3_ for 6 days. After that, the solution was evaporated to dryness, and the concentrated HNO_3_ was added twice to remove fluoride anions and complete digestion. Last, the samples were dissolved in 3.0 M HNO_3_ and then diluted with 2% (v:v) HNO_3_ to 6000 times for major and trace element analyses. Four geological reference materials (AGV-1, BHVO-2, W-2a, and BCR-2) were analyzed and then compared to the published data from (*65*). A standard curve was then established and measured the major and trace element concentrations of the unknown lunar samples. Due to the loss of Si during the sample digestion procedure, SiO_2_ concentration was obtained by subtraction of other major elements from 100 wt %. The relative SD (RSD) of the elements measured by ICP-MS was less than 5%.

### SIMS Pb-isotope analyses

After SEM and EPMA analyses were completed, the mounts were cleaned with an ultrasonic cleaner containing detergent, absolute alcohol, and deionized water. They were then placed in a vacuum oven to dry at 50°C for over 6 hours. In situ Pb-Pb analyses of two baddeleyites in the VLT basalts were carried out using the CAMECA IMS 1280 HR secondary ion mass spectrometer (SIMS) installed at GIGCAS, following the analytical procedures described by (*20*). A Gaussian illumination mode was used to focus a primary beam of ^16^O^−^ to 2 × 3 μm in size, with an intensity of ~330 pA. Five low-noise (<0.003 counts per second) electronic multipliers (EMs) with electronically gated deadtimes of 65 ns equipped in the multicollector system were used to simultaneously measure ions of^208^Pb^+^ (H1), ^207^Pb^+^ (C), ^206^Pb^+^ (L1), and ^204^Pb^+^ (L2). Entrance (60 μm) and exit (150 μm) slits were set to allow a mass resolution power of ~8000 (50% peak height). A nuclear magnetic resonance controller was used to achieve an instrumental drift (∆*M*/*M*) < 1.5 parts per million over 1 hour. Before each measurement, a primary 8-nA beam of ^16^O^−^ was used to remove the gold coating and minimize possible surface contamination. The ion images with ^90^Zr^+^ on a 25 μm–by–25 μm area were used to precisely locate the target minerals. Each measurement consisted of 4 s × 80 cycles, lasting ~9 min. High-purity oxygen flooding was introduced into the sample surface during analysis to effectively improve Pb^+^ sensitivity. Analyses of the National Institute of Standards and Technology (NIST) reference material, SRM 610, were used to calculate the relative yield of different EMs and evaluate the external reproducibility. The reproducibility obtained from the NIST SRM 610 measurements (^208^Pb/^206^Pb = 0.55%; ^207^Pb/^206^Pb = 0.73%; ^204^Pb/^206^Pb = 2.69%, reported as 1 RSD, *n* = 32) and the uncertainties on each unknown analysis were propagated to determine the overall uncertainties of single spot analysis, which are stated in data S1. Within uncertainty limits, no systematic drift was observed in the NIST SRM 610 measurements during a given analytical session (complete dataset presented in data S1). Phalaborwa baddeleyite standard [^207^Pb^*^/^206^Pb^*^ = 0.1272; (*66*)] as the unknown sample was measured to monitor the correction factor and data processing.

### In situ Rb-Sr isotopic analyses by LA-MC-ICP-MS

In situ Rb-Sr isotopic analyses in this study were performed on an MC-ICP-MS (Neptune Plus, Thermo Scientific), coupled with a nanosecond193-nm laser ablation system (RESOlution M-50, Resonetics), hosted at SKLaBIG, GIGCAS. The laser parameters were set as follows: beam diameter of 82 to 155 μm, repetition rate of 10 Hz, and energy density of ~4 J cm^−2^. Each analysis consisted of 450 cycles with an integration time of 0.131 s per cycle. The first 30 s was used to detect the gas blank and then followed by 30-s laser ablation for sample signals collection. The interferences of Kr on Sr were corrected by subtracting gas blank from the raw signal intensities. ^85^Rb was used to correct the interference of ^87^Rb on ^87^Sr with a natural ^85^Rb/^87^Rb = 2.593 (*67*). Although some studies observed the interferences of Ca dimers (e.g., ^43^Ca^44^Ca^+^) and Ca argides (e.g., ^44^Ca^40^Ar^+^) during Sr isotope measurements on carbonates [e.g., (*68*)], other studies suggest a very limited role for interfering Ca dimers and Ca argides [e.g., (*69*)], which was also confirmed on the instrument used in our experiment (*70*). The mass bias of ^87^Sr/^86^Sr was normalized to ^86^Sr/^88^Sr = 0.1194 with an exponential mass fractionation law. ^87^Rb/^86^Sr was calibrated with the standard-sample-bracketing method, and the calibration factor was derived from the measurement of a geological reference glass (BCR-2G). The detailed data reduction procedure is reported in (*70*). Seven analyses of PZHPL plagioclase (An = 56) and five analyses of MKAn plagioclase (An = 96) yielded a weighted mean of ^87^Sr/^86^Sr = 0.70437 ± 0.00009 (2 SD) and 0.70347 ± 0.00011 (2 SD), respectively, which agree within error with the result measured by solution MC-ICP-MS and thermal ionization mass spectrometry method (*71*).

### MSP piston-cylinder experiments

MSP experiments were conducted at SKLaBIG, GIGCAS, to evaluate our methods for estimating the formation P-T of the CE-6 basalts. We performed 12 experiments on a Rockland piston-cylinder apparatus at conditions between 0.4 and 1.3 GPa and between 1100° and 1230°C (table S5 and fig. S6A), with the starting composition synthesized on the basis of average of CE-5 basalts (*10*). The experiments were conducted on CE-5 composition for comparison given the following considerations: (i) The CE-5 basalts and CE-6 basalts have similar major element compositions. (ii) As melts that may have primarily been derived from an IBC source, the CE-5 basalts are relatively more primitive with higher Mg# (~33 to 34) than the CE-6 basalts (~31 to 32). (iii) The purpose of the experiments is to check whether MSP yielded by modeling and the results of mineral thermobarometers are valid or not. For the CE-5 basalts, the latter has already been published and can thus be used directly for comparison. The P-T results obtained using our methods (MSP modeling and mineral thermobarometers) were then compared to the experimental run conditions (Supplementary Text). The validated methods were then applied to the CE-6 basalts.

The starting material was synthesized by first weighting and mixing reagent oxides (SiO_2_, TiO_2_, Al_2_O_3_, MnO, MgO, Cr_2_O_3_, NiO, and P_2_O_5_) or carbonates (CaCO_3_, Na_2_CO_3_, and K_2_CO_3_), and then homogenizing the mixture by grinding in an agate mortar for 2 hours. The homogenized mixture was then decarbonated in a Pt crucible in a muffle furnace at 1000°C overnight, and, then, iron was added to the decarbonated mixtures as FeO and Fe_2_O_3_ at a ratio of 97.5:2.5, which was used to adjust the oxygen fugacity at around iron-wüstite (IW) buffer, consists with the assumption for the lunar basalt source [∼ΔIW −0.5; (*72*, *73*)].

For each sample, ~20 mg of the starting material was packed into a graphite-lined platinum capsule (3-mm outer diameter, 2.7-mm inner diameter, and 8-mm length), dried overnight in an oven, and then welded shut. The welded sample was placed into a ^3^/_4_-inch (1.905 cm) assembly, which comprises of MgO inserts, graphite heater, Pyrex glass, and pyrophyllite tube. The friction correction of this assembly was −−22% as estimated by the melting point of CsCl. The pressure uncertainty is <0.1 GPa as estimated from the friction correction of the assembly. The experiment was first heated to above liquidus, maintained for 2 hours, cooled to the final temperature at a rate of 5°C/hour, and then maintained at the target temperature for 48 hours. After the experiment, the recovered sample was cut into two halves, embedded in epoxy resin, and polished for optical observation and EPMA analysis.

## Supplementary Materials

### The PDF file includes:

Supplementary Text Figs. S1 to S10 Tables S1 to S5 Legends for data S1 to S5 References
