# Supplementary material for: The source and thermal driver of young (<3.0 Ga) lunar volcanism
Source: Sci Adv. 2025 Aug 22;11(34):eadv9085. doi: 10.1126/sciadv.adv9085 (PMC12372884; doi:10.1126/sciadv.adv9085)
Supplement: Supplementary file 1 — Supplementary Text Figs. S1 to S10 Tables S1 to S5 Legends for data S1 to S5 References [file sciadv.adv9085_sm.pdf]

Science Advances
AAAS

Supplementary Materials for

The source and thermal driver of young (<3.0 Ga) lunar volcanism

Chengyuan Wang *et al.*

Corresponding author: Yi-Gang Xu, yigangxu@gig.ac.cn

Sci. Adv. **11**, eadv9085 (2025)
DOI: 10.1126/sciadv.adv9085

The PDF file includes:

Supplementary Text
Figs. S1 to S10
Tables S1 to S5
Legends for data S1 to S5
References

Other Supplementary Material for this manuscript includes the following:

Data S1 to S5

Supplementary Text

Modeling of the melt evolution

Variation of TiO_2 in CE-6 basalts was calculated from the compositions of pyroxenes in CE-6 samples (Fig. 2A). Partition coefficient of Ti in pyroxene ($D_{Ti}^{Pyx/melt}$) was calculated according to the linear function of their Ca contents of pyroxenes: $D(TiO_2) = 0.0148 \times (CaO_{pyx}) + 0.09 \pm 0.05$ (*80*). We selected the high partition coefficients to estimate the minimum TiO_2 content in melts. We used a fixed pyroxene-liquid Fe-Mg exchange coefficient (Kd) of 0.28 at temperature of ~1200 °C (*36*). The average of TiO_2 content in melts was calculated for an increment of Mg# = 1 to 5.

The evolution processes of the CE-6 LT and VLT basalts were also modeled by the PETROLOG program (*25*). The bulk compositions of the CE-6 LT and VLT basalts were used as parental melts. All modeling assumed pure fractional crystallization (100%) at a constant pressure. The oxygen fugacity was set at IW buffer. Mineral solution models used include olivine (*81*), clinopyroxene (*82*), orthopyroxene (*81*), plagioclase (*82*), and ilmenite (*83*). The pressure of near-surface fractionation was set at 1 kbar, considering negligible differences among the modeling results when pressures are less than 1 kbar.

Fractional crystallization modeling

The modeling of Ta/Nd and Zr/Nb ratios during fractional crystallization of clinopyroxene from mare basalt (Fig. 3C) was calculated using the equation $C_l/C_0 = (1-F)^{D-1}$, where C_l = concentration of an element in the final melt, C_0 = concentration of an element in the parental melt, F = weight fraction of crystallized crystals, D = bulk partition coefficient. Trace element compositions of the Apollo LT (12064) were used for the parental melt. The Apollo LT (12064) is selected since its main crystallizing phases are clinopyroxene (~56 vol.%), plagioclase (~29 vol.%), ilmenite (~4 vol.%) and mesostasis (~9 vol.%), with scarce olivine (*84*). This is similar to the mineral phases in the CE-6 LT basalts, and is suitable for modeling of clinopyroxene fractionation. The average of D for clinopyroxene from (*28*) was used.

Modeling of non-modal mantle melting

We employed a non-modal melting model to investigate the influence of varying proportions of IBC (ilmenite-bearing cumulate) components in the source on the generated partial melts. The calculation results were compared with measured compositions of lunar basalts and illustrated in Fig. 3C. The IBC is primarily composed of clinopyroxene and ilmenite, and their average trace element compositions are from (*30*). Because clinopyroxene and ilmenite host the majority of HFSE (high field-strength elements) in the lunar mantle (HFSE are extremely incompatible in olivine and orthopyroxene), their compositions were used to calculate the source compositions with varying proportions of these two minerals (Fig. 3C). The variations of Ta/Nd and Zr/Nb ratios in the partial melts were calculated by a non-modal melting equation: $C_l/C_0 = 1/(D + F \times (1 - P))$, where C_l = concentration of an element in the melt, C_0 = concentration of an element in the mantle source, F = weight fraction of melt produced, D = bulk partition coefficient, and $P = \sum_\alpha D^i_\alpha \times P_\alpha$, with D^i_α = partition coefficient of element i in phase α, and P_α = proportion of mineral phase entering liquid. P_α was obtained from the melting equation of the IBC formulated by the experimental study: 0.67 clinopyroxene + 0.33 ilmenite → 1 melt (*29*). The partition coefficients for clinopyroxene and ilmenite are the same as in fractional crystallization modeling. The calculation details are given in data S5.

Estimating P-T conditions of the CE-6 basalt sources

Before the estimation, we firstly used the results of multiple saturation point (MSP) experiments to evaluate the validity of our methods (MSP modeling and mineral thermobarometry). The starting composition is synthesized based on the average of CE-5 basalts (*10*). We performed twelve experiments at conditions between 0.4 to 1.3 GPa and 1100 to 1230 °C (table S5 and fig. S6A). At 0.4 GPa, plagioclase and olivine are liquidus phases within ~20 °C of the liquidus, while clinopyroxene and ilmenite are observed to crystallize at 1100 °C. At high pressure (≥ 1 GPa), clinopyroxene is the only near liquidus phase. Garnet and ilmenite present at 1.3 GPa when temperature decreases to 1170 °C (fig. S10A), which was also observed in (*14*). The presence of garnet at 1.3 GPa precludes the deep derivation of the melt; otherwise, trace element compositions would be influenced (i.e., high Sm/Yb) by the garnet fractionation. At 0.7 GPa, both plagioclase and clinopyroxene appear as near-liquidus phases (fig. S10B), while ilmenite crystallizes at 1110 °C without olivine. These results indicate that the liquidus for CE-5 basalts is multiply saturated with clinopyroxene, plagioclase and probably olivine between 1140–1160 °C and at ~0.6 GPa (fig. S6A). We then used the program Geo-Pseudo (*38*) to simulate the same process. The result yields MSP (1130–1150 °C, ~0.55 GPa) in good agreement with the experiments (fig. S6B), thereby assuring the validity of this method in estimating MSP P-T conditions. We also modeled the MSP of Apollo picritic glass and basalts using this program (fig. S6C, D). The good match between the modeled results and those produced by experiments for the Apollo mare basalts proves the reliability of this method.

We then evaluated the validity of different thermobarometers using the compositions of minerals formed in the experiments (data S3). Using the compositions of plagioclase and glass in the experiments, we found that the thermometers all yield relatively higher temperatures than the experimental run conditions, while the plagioclase-liquid thermometer (Eq. 26) (*40*) yields the best estimates to experimental conditions (fig. S6E). The clinopyroxene-liquid barometer (*39*) is better than the clinopyroxene-only barometer (*85*), since it yields more consistent results in comparison with the experimental pressures (fig. S6F).

We suppose that the deviation of some thermobarometers is caused by the following reasons: 1) The plagioclase-liquid thermometer (Eq. 23, 26) in (*40*) was given according to the experiments with conditions set for earth volcanism. At low temperatures (i.e., < 1200 ℃), most of the datasets to constrain the thermometer are from hydrous experiments, whereas mare basalts were formed in anhydrous conditions; 2) The pressure given by clinopyroxene-only barometer in (*85*) is also shown to be underestimated with low water content; 3) The clinopyroxene-liquid compositions in (*39*) are mainly used for barometer, which is valid in this study. However, the temperatures they provide are calculated using their barometer in concert with the thermometer in (*40*) and would be affected by the same problem from the datasets of hydrous experiments.

The proven methods were then applied to the CE-6 basalts. As discussed in the main text, we used the bulk compositions of the CE-6 LT and VLT basalts as proxies for the primary magma. These compositions were then subjected to equilibrium crystallization simulations using the Geo-Pseudo program (*38*) over a range of pressure and temperature (0–12 kbar and 1000–1300 °C, respectively) (Fig. 4), which employs an internally consistent thermodynamic dataset (*86*). The Fe_2O_3 content was set as zero (fO_2 < IW). The ultramafic solid solution model of clinopyroxene, olivine, spinel, and melt from (*87*) was used in the calculation. Note that ilmenite is the late-stage crystallization phase (see discussion) and is not shown in the diagram.

For the clinopyroxene-liquid barometric calculation (*39*), only magnesian clinopyroxenes (Mg# > 60) were selected for calculations (data S2). Clinopyroxenes with cation sums of <3.98 or >4.02 and very low Na and Al contents (Jd < 0.002, total Al < 0.11) were excluded. Clinopyroxenes in the porphyritic LT basalts were not used as they were formed during rapid

crystallization (see main text for discussion). The plagioclase-liquid thermometer (Eq. 26) (*40*) was applied using the estimated pressure and plagioclase data with An > 90 (plagioclases in the porphyritic LT basalts were not used either) (data S2).

Modeling of variation in thermal gradient related to magma underplating

We use a simplified one-dimensional heat flow equation to model the heat conduction from underplated melts (Equation 1). For the Apollo basin, the initial state of crustal geotherm is set with a surface temperature of 0 °C, crustal thickness of 25 km (*17*), and a surface heat flow of 58 mW/m^2 based on the estimation of surface heat flow in the non-PKT region (*64*). Since the ~3.17 Ga low-Ti picritic basalt 12002 has an MSP with olivine and pyroxene at 1.25 GPa (~250 km) and 1380 °C (*11, 88*), we set the base of lithosphere at ~3 Ga at a depth of 250 km and temperature of 1290 °C (fig. S8A), slightly lower than peridotite solidus at this pressure. When magma underplating occurred, we assumed a 10 Ma pulse of melt flux emplaced at a constant rate below the rheological trap and modified the geotherms above. The modeling results are given in fig S8. For the underplating at the lithospheric base (fig. S8B, rheological trap 1) and IBC base (fig. S8C, D, rheological trap 2), we set the depth of underplated melts at 250 km and 120 km, respectively.

$$\rho C_p \frac{\partial T}{\partial t} = k \cdot \frac{\partial^2 T}{\partial y^2} + H \qquad (1)$$

In Equation 1, T is temperature, ρ is density (crust: 2900 kg/m^3; IBC: 3500 kg/m^3; peridotite: 3400 kg/m^3) (*54, 89*), C_p is heat capacity (1000 J/(kg·K) for both crust and mantle) (*54*), t is time, y is depth, k is thermal conductivity (crust: 1.5 W/(m·K); mantle: 4 W/(m·K)) (*54, 90*), and H is the heat flow (W m^{-2}) produced by underplated melts (Equation 2).

$$H = F\rho C_p \cdot \Delta T/\Delta t \qquad (2)$$

In Equation 2, F is the underplating flux (km/Ma) in 1 m^2 area, ρ is density of melt (3010 kg/m^3) (*57*), C_p is heat capacity of melt (1250 J/(kg·K)), ΔT is assumed to be 400 °C (temperature difference between mantle potential temperature of ~1440 °C (*26*) and basaltic solidus of ~1040 °C at ~5 kbar (Fig. 4)), Δt is 1 Ma. For underplating flux of 0.2 km/Ma, the produced heat flow is calculated to be ~10 mW/m^2, similar to or somewhat lower than the ~24 mW/m^2 given in (*91*).

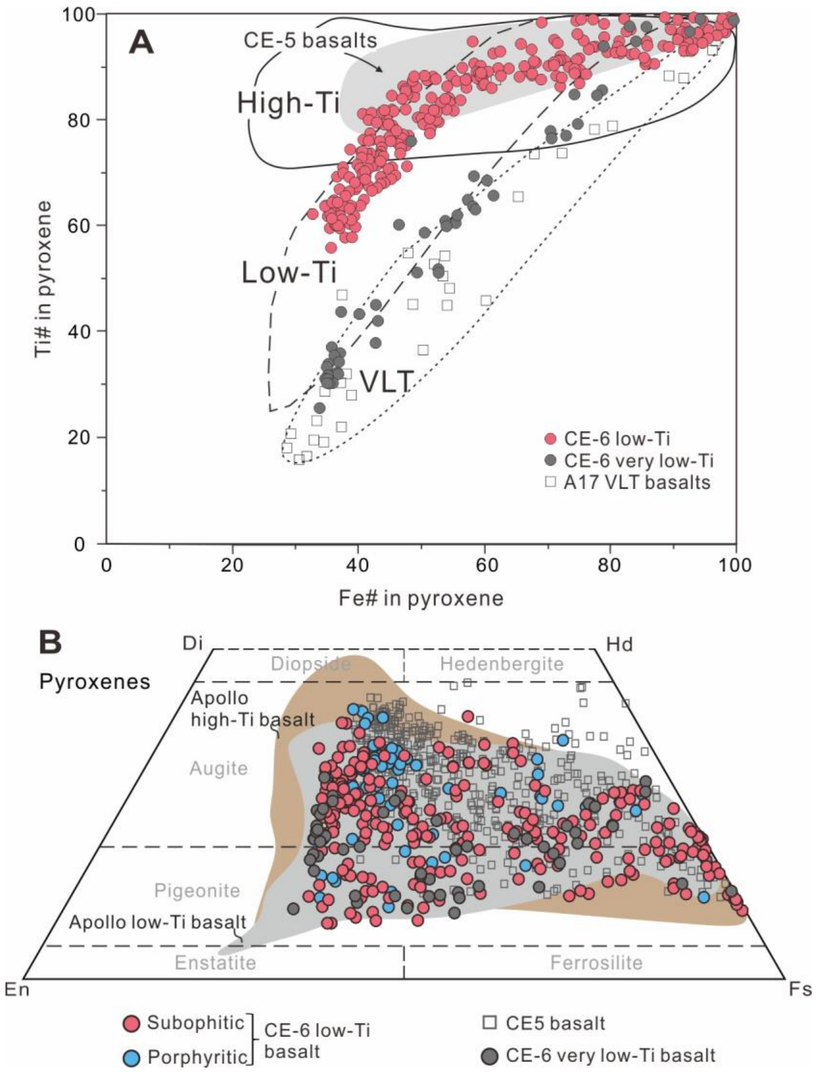

Fig. S1. Compositions of pyroxene in the Chang'e-6 basalts.

(A) Ti# [100*molar Ti/(Ti + Cr)] versus Fe# [100*molar Fe/(Fe + Mg)] for pyroxene from CE-6 basaltic clasts. The fields of CE-5, and Apollo high-Ti, low-Ti (LT) and very low-Ti (VLT) basalt data are taken from (*10*), Astromaterials Data System (https://www.astromat.org/) (*92*) and (*93*), respectively. **(B)** Comparison of pyroxenes in CE-6 LT and VLT basalts with those of the CE-5 (*8, 10*) and Apollo mare basalts (*16*).

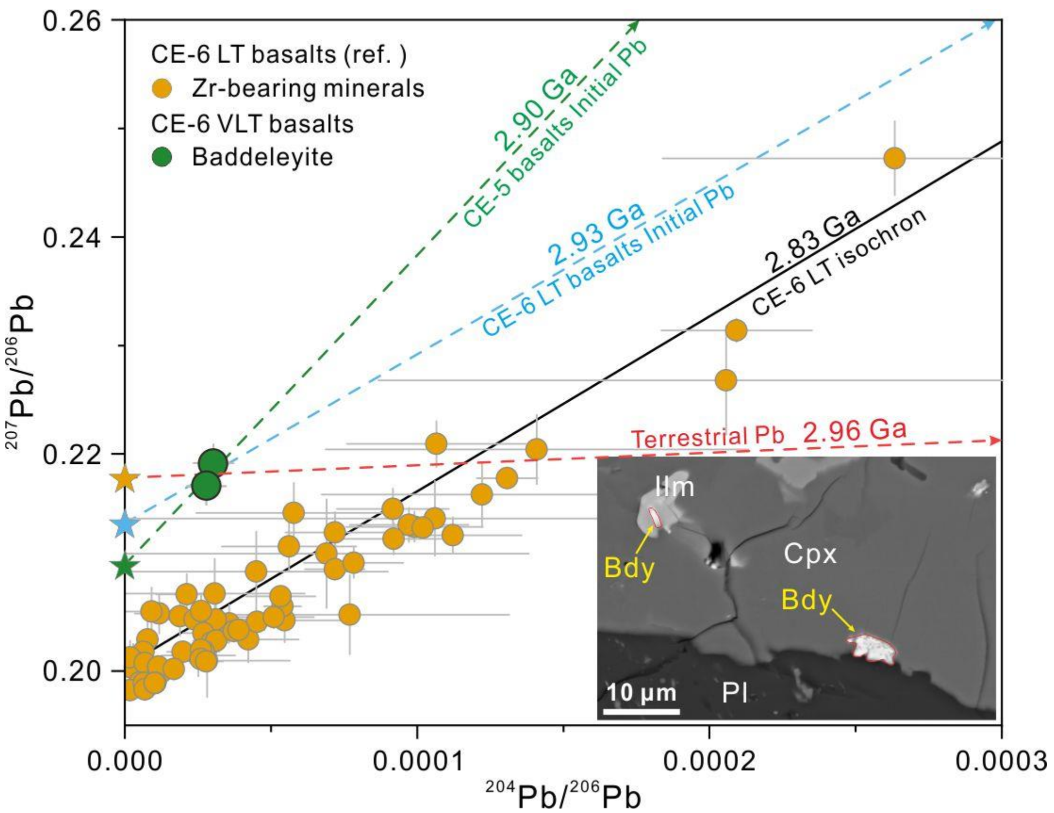

Fig. S2. $^{207}Pb/^{206}Pb$ vs. $^{204}Pb/^{206}Pb$ diagram for dated baddeleyites in CE-6 VLT basalts. The VLT data show an obvious departure from the ~2.83 Ga isochron defined by the CE-6 LT basalts, meaning that the two suites of basalts were not formed at the same time. Dashed lines show modeled results considering the influence of terrestrial and lunar common Pb (table S2). The black line shows isochron of CE-6 LT basalts from (*20, 22*). Uncertainties are portrayed at the 1s level.

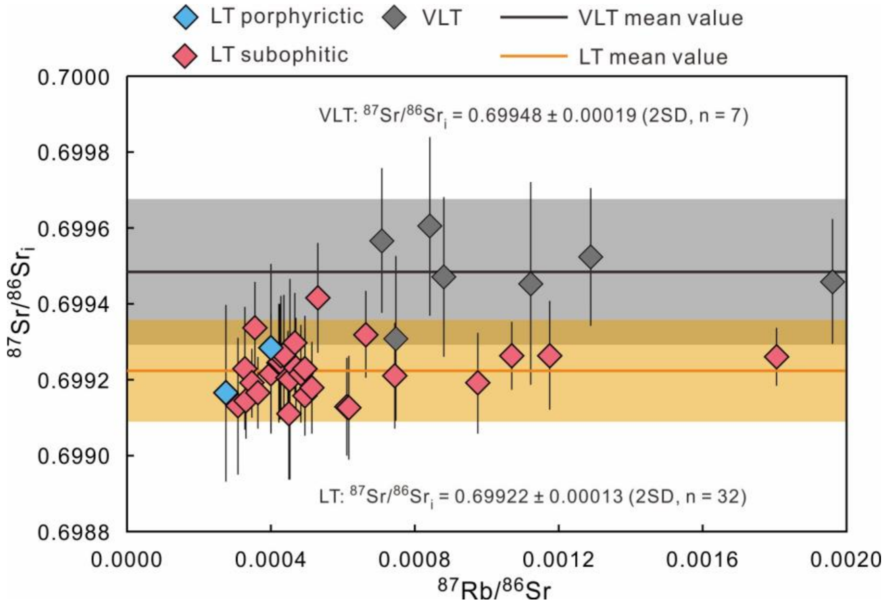

Fig. S3. Initial $^{87}Sr/^{86}Sr$ vs. $^{87}Rb/^{86}Sr$ diagram for plagioclases in CE-6 LT and VLT basalts. Error bars represent 2σ.

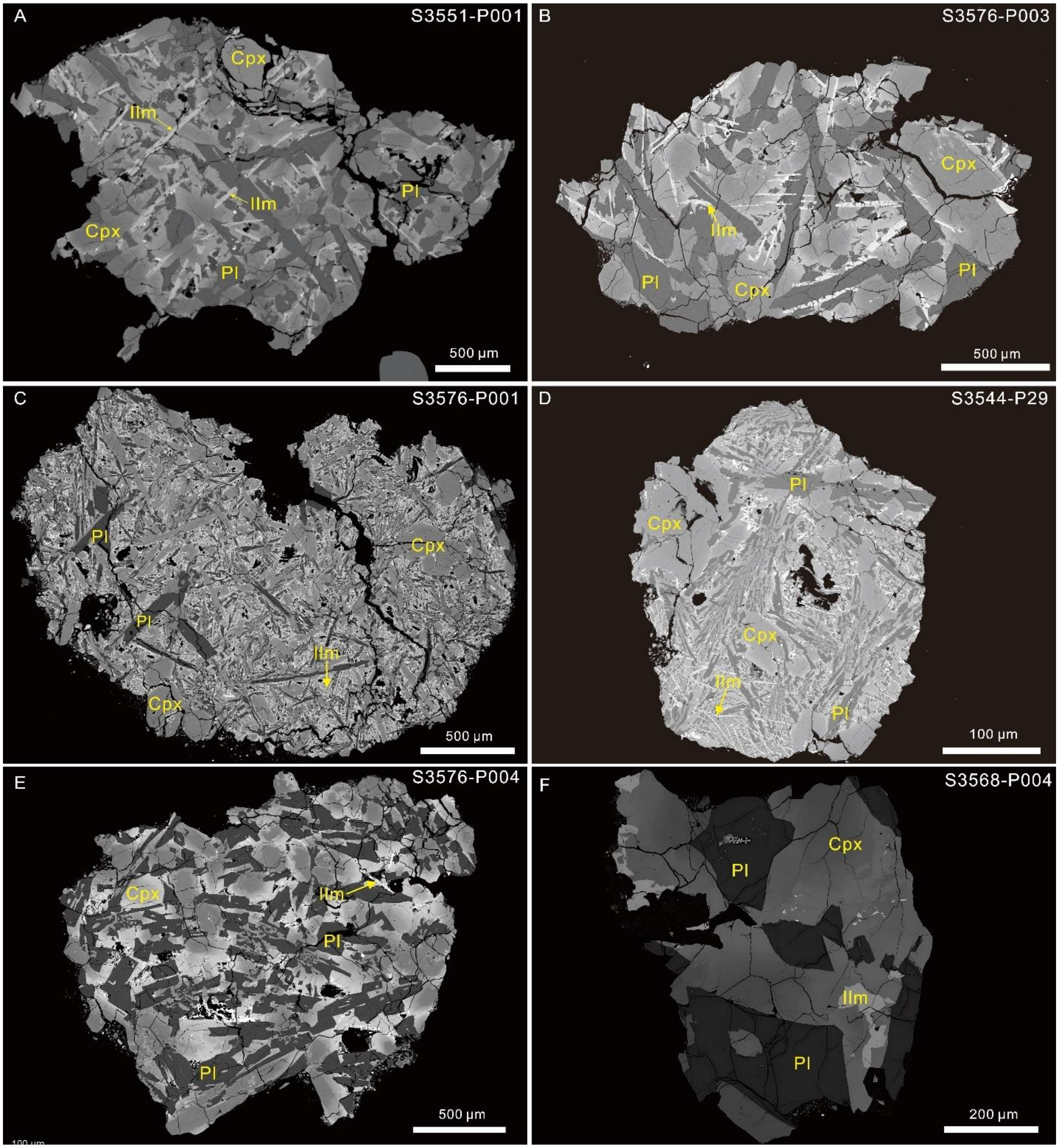

Fig. S4. Representative backscattered electron (BSE) images of the CE-6 LT and VLT basaltic clasts.

(A-B) Subophitic LT basalt. **(C-D)** Porphyritic LT basalt. **(E-F)** Porphyritic VLT basalt. Pl-plagioclase, Cpx-clinopyroxene, Ilm-ilmenite.

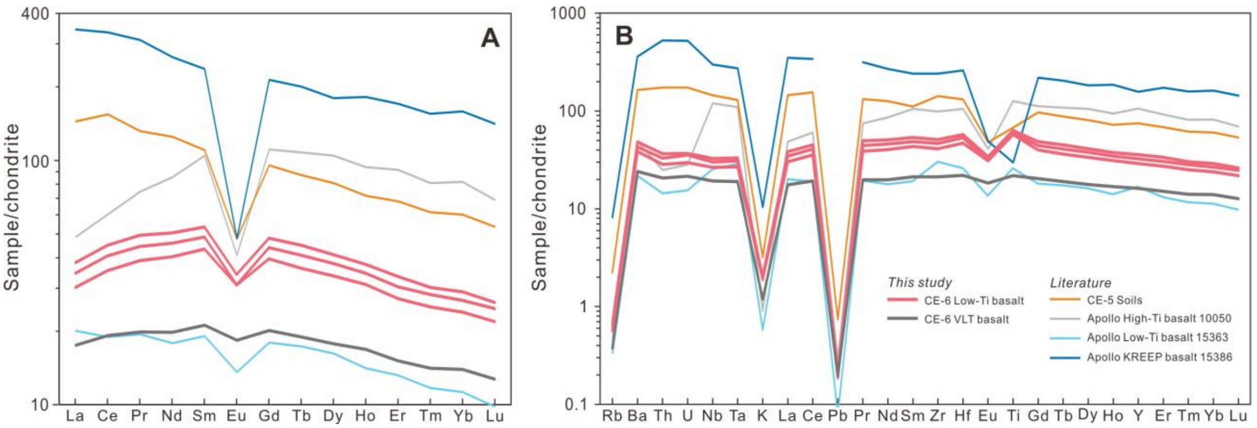

Fig. S5. Trace element compositions of the bulk CE-6 LT and VLT basalts.

(A) Chondrite-normalized REE and **(B)** trace element distribution patterns of CE-6 low-Ti basalt. The CE-5 soils, Apollo high-Ti (10050), low-Ti (15363) and KREEP basalts (15386) data are taken from (*76*), (*94*), (*95*), and (*78*) respectively. The chondrite is from (*96*).

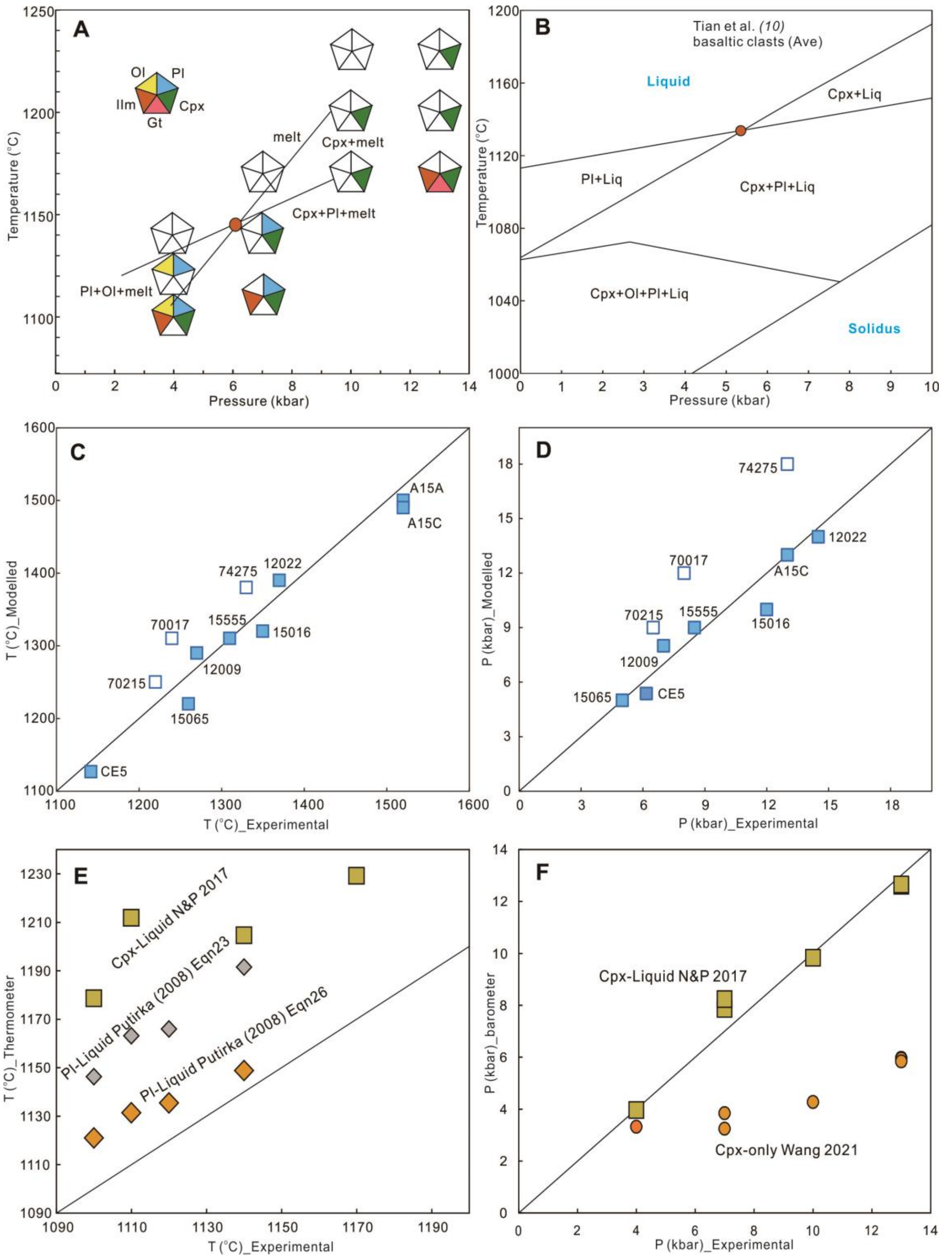

Fig. S6. Comparison between the piston-cylinder experimental results, MSP modeling, and thermobarometric data.

(A) Experimental results in the pressure-temperature diagram. The red dot denotes the MSP in the diagram. Stable mineral phases are depicted in pentagons at the individual P-T conditions: clinopyroxene (Cpx), olivine (Ol), plagioclase (Pl), garnet (Gt), and ilmenite (Ilm). **(B)** Phase

equilibrium calculations of the CE-5 basalts constructed by the Geo-Pseudo program. **(C-D)** P-T of the multiple-saturation points for CE-5 basalts (*10*) and Apollo lunar materials calculated using the Geo-Pseudo program, compared with experimental results of this study and (*97*). Filled squares represent data of low-Ti basalts and picritic glass. Unfilled squares represent data of high-Ti basalts, for which the pressure could be overestimated by the program. **(E-F)** Comparison of P-T estimates by different thermobarometers (*39, 40, 85*) using the compositions of minerals formed in the experiments with the experimental run conditions.

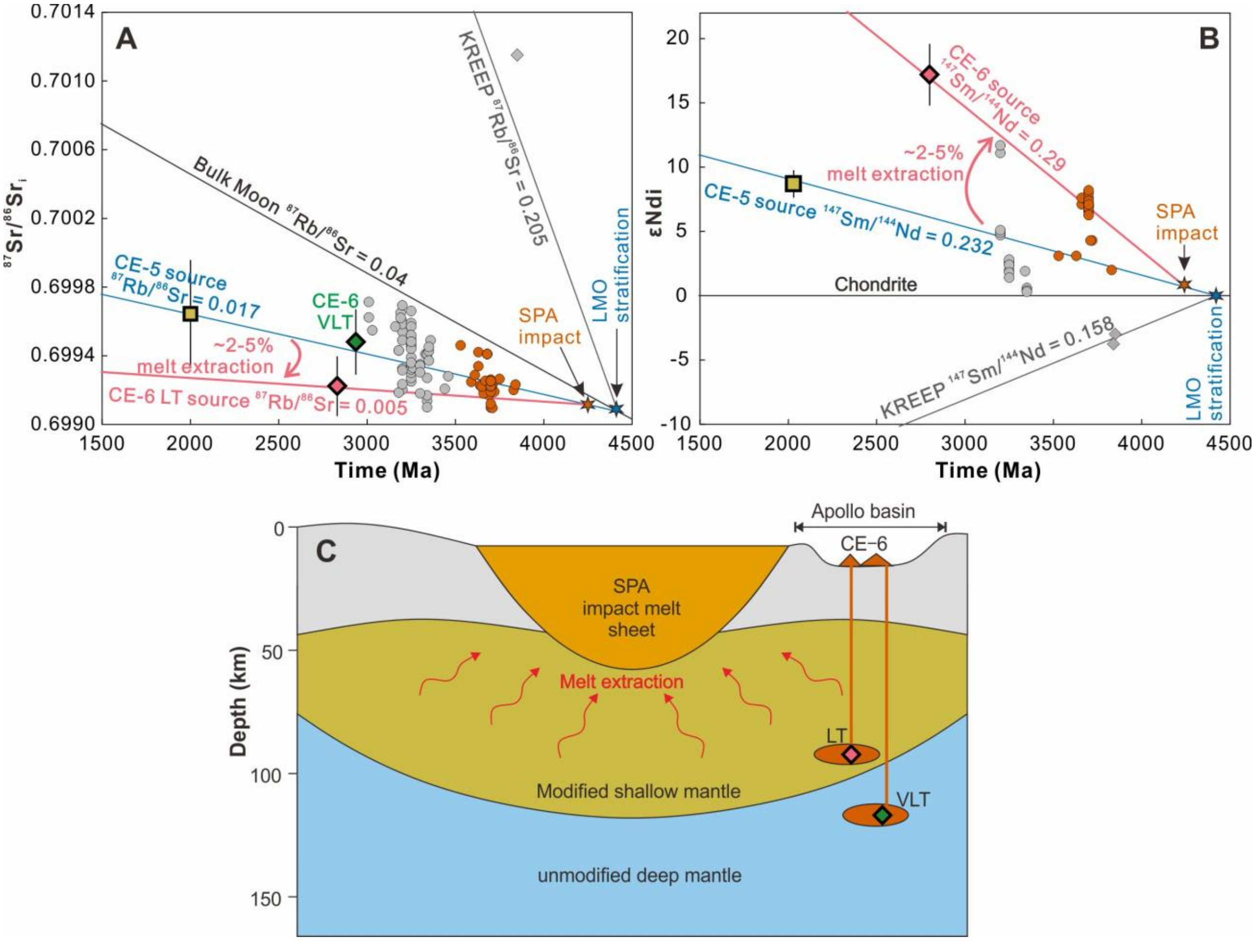

Fig. S7. Influence of the SPA impact on the underlying mantle.

Variation of initial $^{87}Sr/^{86}Sr$ **(A)** and εNd **(B)** of the lunar basalts with time. Symbols are same as in Fig. 3. Labels on the lines denote $^{87}Rb/^{86}Sr$ and $^{147}Sm/^{144}Nd$ of the sources (*20*). The error bars are 2δ. Data sources are as in (*20, 98*). Timing of the SPA impact and LMO stratification are respectively taken from (*48*) and (*2*). Degree of melt extraction is calculated using the CE-5 source (*10*) as unmodified IBC, following batch-melting model from (*99*). **(C)** Proposed scenario for the modification of mantle beneath SPA and Apollo basin by the SPA impact.

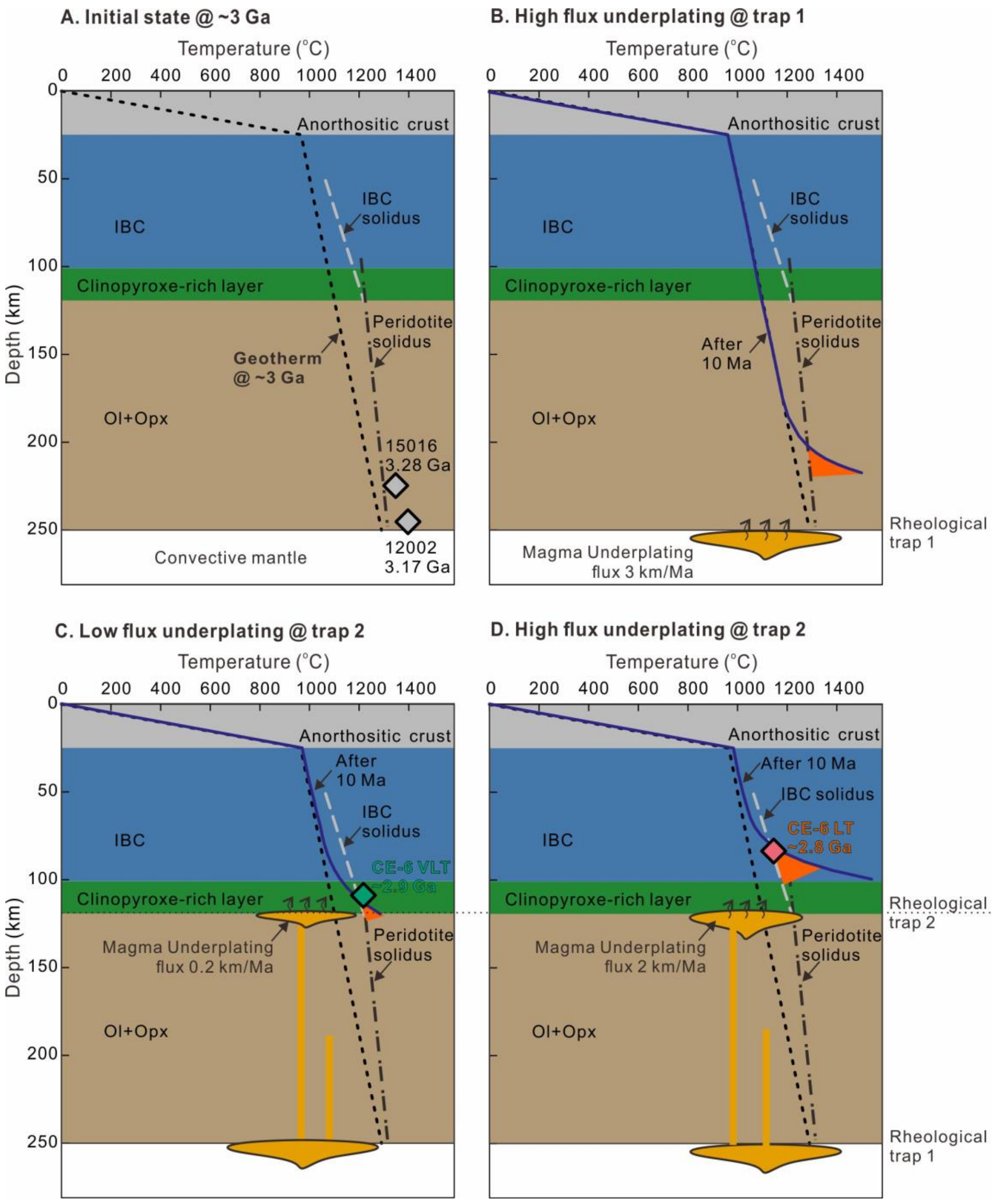

Fig. S8. Modeling of variation in thermal gradient related to magma underplating

(A) The initial state of geotherm (black dashed line) at ~3 Ga. The age and MSP of 12002 and 15386 are from (*11, 88, 100, 101*). The peridotite solidus T (℃) = 1409+134.2P-6.581P^2+0.1054P^3-273 is from (*102*), P is pressure (GPa). The IBC solidus is from (*34*) (1130 ℃ at 80 km and 1220 ℃ at 120 km). The crust-mantle structure is modified from Fig. 5B. **(B-D)** Variation of geotherms at different underplating flux at rheological trap 1 and 2. The orange lines depict modified geotherms after a pulse of magma underplating for 10 Ma. Note that thermal conduction from underplated melts at the lithosphere base is incapable of inducing melting of IBC. Melting of IBC is only possible when magmas were trapped at the base of the IBC layer **(C, D)**.

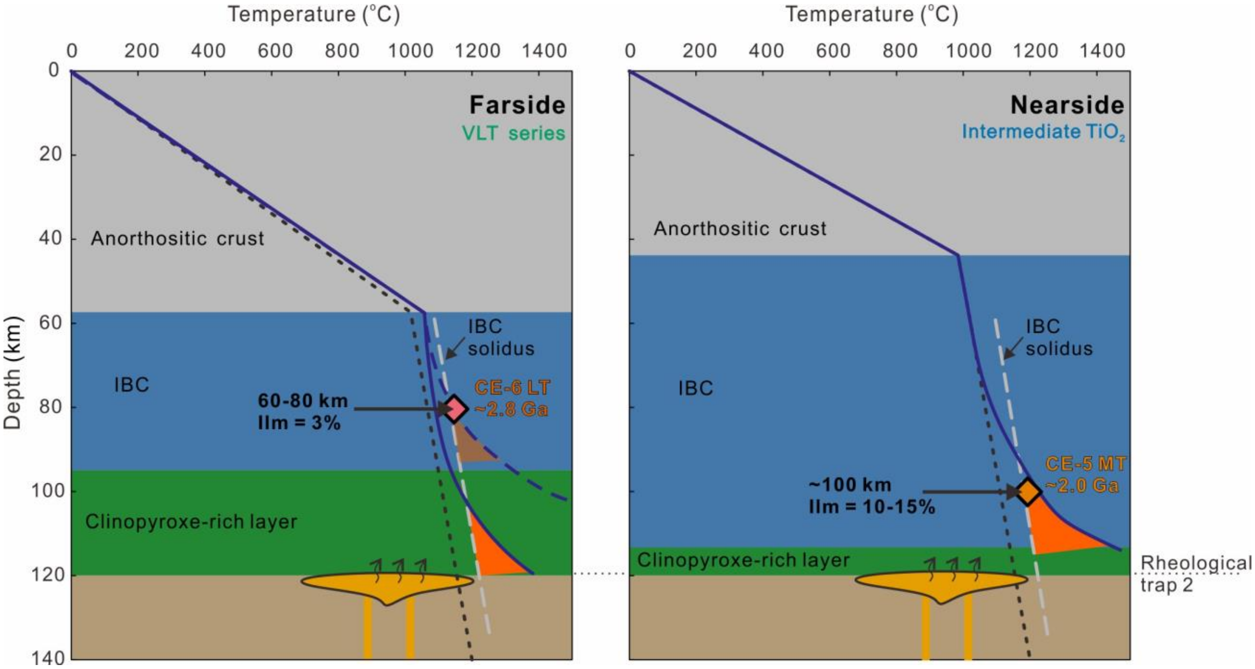

Fig. S9. Asymmetric composition and thickness of IBC in the uppermost mantle.

Different composition and thickness of IBC in the uppermost mantle of the nearside and farside leads to different secular changes in TiO_2 of young mare basalts from each hemisphere (Fig. 5C). In the farside hemisphere, the IBC is thin and contains less ilmenite, ensuring production of predominant VLT basalt. In the nearside hemisphere, melting of the thick and ilmenite-rich IBC due to thermal conduction from magmas underplated at the base of the IBC could produce intermediate TiO_2 mare basalts, as represented by the CE-5 basalts. The source information of CE-6 LT and CE-5 intermediate-TiO_2 basalts is from Fig. 3, 4 and (*16*), respectively.

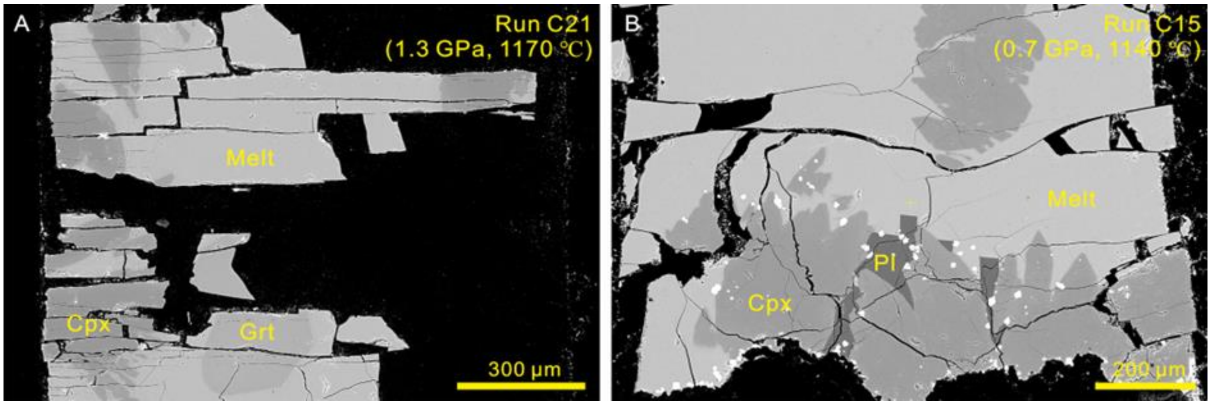

Fig. S10. Representative backscattered electron (BSE) images of the run products of piston-cylinder experiments.

(A) BSE image of Run C21 (1.3 GPa, 1170 ℃); **(B)** BSE image of Run C15 (0.7 GPa, 1140 ℃). Pl-plagioclase, Cpx-clinopyroxene, Grt-Garnet.

Table S1.

Rb-Sr isotopic data of clean plagioclase in the CE-6 VLT basalts.

Sample	$^{87}Rb/^{86}Sr$	2SE	$^{87}Sr/^{86}Sr$	2SE	$^{87}Sr/^{86}Sr_i$ (t=2.93 Ga)
S3576-P04-01	0.00071	0.00017	0.69960	0.00019	0.69957
S3576-P04-02	0.00075	0.00013	0.69934	0.00022	0.69931
S3576-P04-03	0.00084	0.00015	0.69964	0.00023	0.69960
S3576-P04-04	0.00088	0.00054	0.69951	0.00021	0.69947
S3576-P04-05	0.00112	0.00031	0.69950	0.00027	0.69945
S3576-P04-06	0.00129	0.00018	0.69958	0.00018	0.69952
S3576-P04-07	0.00196	0.00016	0.69954	0.00016	0.69946
Standard	$^{87}Rb/^{86}Sr$	2SE	$^{87}Sr/^{86}Sr$	2SE	
PZHPl-01	0.020	0.00048	0.70435	0.00007	
PZHPl-02	0.037	0.00035	0.70444	0.00009	
PZHPl-03	0.029	0.00032	0.70433	0.00009	
PZHPl-04	0.024	0.00022	0.70433	0.00008	
PZHPl-05	0.054	0.00061	0.70438	0.00010	
PZHPl-06	0.047	0.00080	0.70441	0.00012	
PZHPl-07	0.035	0.00025	0.70433	0.00009	
mean			0.70437		
2SD			0.00009		
reference value			0.704348 ± 0.000015		
MKAn-01	0.000	0.00002	0.70352	0.00012	
MKAn-02	0.000	0.00002	0.70344	0.00011	
MKAn-03	0.000	0.00002	0.70345	0.00012	
MKAn-04	0.000	0.00002	0.70354	0.00011	
MKAn-05	0.000	0.00002	0.70342	0.00012	
mean			0.70347		
2SD			0.00011		
reference value			0.70345 ± 0.000020		

Table S2.

Modeled influence of common lead corrections on the measured Pb-Pb age for baddeleyite grains in CE-6 VLT basalts. The influences of terrestrial common Pb (0 Ma), lunar common lead introduced to the baddeleyite at the time of crystallization (~2.9 Ga) from basalt sources, were evaluated. The Sr isotope of VLT basalts falls within the CE-5 basalt (μ2 = 684) and the CE-6 LT basalt (μ2 = 355) scope, which is the reason that the range of μ2 values was selected. In all scenarios, a maximum shift in the Pb-Pb age of 57 Ma is observed, showing that the influence of common lead on the calculated age is less than 2%. The mean of all scenarios results of 2936 ± 26 Ma (1σ) is interpreted as the crystallization age of the CE-6 VLT basalts.

SIMS measured results						*SK 0 Ma Terrestrial*		*μ2 = 355*		*μ2 = 684*	
					$^{206}Pb/^{204}Pb$	18.703		167.989		298.725	
					$^{207}Pb/^{206}Pb$	0.8356		1.1206		1.082	
Sample spot #	*$^{206}Pb/^{204}Pb$*	*Error (%)*	*$^{207}Pb/^{206}Pb$*	*Error (%)*		*Corrected $^{207}Pb/^{206}Pb$ age ± 1σ (Ma)*					
S3568 004 Bdy@01	3.03E-05	23	0.21915	0.78		2972	14	2940	24	2915	33
S3568 004 Bdy@02	2.79E-05	24	0.21705	0.79		2957	14	2927	24	2904	33
					ave.			2936			
					1 σ			26			

Table S3.

Bulk major element compositions in CE-6 LT and VLT basalts measured by solution ICP-MS. Data of the CE-5 basalts are shown for comparison.

Sample	S3551-P001		S3576-P001		S3576-P003		S3576-P004		CE-5*	
Note	Low-Ti	1SD	Low-Ti	1SD	Low-Ti	1SD	Very low-Ti	1SD	Low-Ti	1SD
Major elements (wt. %)										
SiO_2	47.3	0.34	46.4	0.25	44.2	0.22	43.3	0.22	41.3	1.52
TiO_2	4.54	0.07	4.37	0.07	4.83	0.04	1.67	0.02	5.12	0.14
Al_2O_3	8.78	0.07	10.8	0.11	9.42	0.09	12.9	0.08	11.6	0.22
FeO	22.2	0.27	20.4	0.18	23.1	0.16	21.8	0.04	22.7	0.90
MnO	0.28	0.004	0.26	0.002	0.29	0.003	0.29	0.003	0.28	0.01
MgO	5.61	0.08	5.37	0.06	5.80	0.05	6.40	0.06	6.52	0.29
CaO	10.6	0.17	11.7	0.08	11.6	0.10	12.9	0.19	11.6	0.30
N_2O	0.33	0.005	0.39	0.01	0.35	0.003	0.33	0.004	0.46	0.01
K_2O	0.13	0.002	0.12	0.002	0.13	0.001	0.08	0.001	0.21	0.01
Cr_2O_3	0.27	0.004	0.21	0.002	0.24	0.002	0.27	0.002		
Mg#	31.3	0.40	32.2	0.33	31.1	0.24	34.6	0.21	33.9	0.62

* Data are taken from (*76*).

Table S4.

Bulk trace element compositions of CE-6 LT and VLT basalts measured by solution ICP-MS.

Sample	S3551-P001		S3576-P001		S3576-P003		S3576-P004	
Note	Low-Ti	1SD	Low-Ti	1SD	Low-Ti	1SD	Very low-Ti	1SD
Trace elements (ppm)								
Co	30.7	0.31	27.2	0.41	29.6	0.33	32.7	0.39
Ni	25.0	0.35	22.6	0.14	15.9	0.37	34.5	0.41
Rb	1.49	0.03	1.33	0.01	1.60	0.03	0.89	0.02
Sr	229	2.06	247	3.46	251	1.25	161	1.29
Y	50.5	0.71	45.3	0.14	55.9	0.34	25.2	0.20
Zr	180	2.16	158	1.11	196	1.18	81.7	0.65
Nb	7.43	0.07	6.55	0.09	8.06	0.04	4.76	0.05
Ba	103	0.93	92.9	0.65	116	1.16	58.0	1.04
La	8.46	0.10	7.40	0.07	9.35	0.09	4.30	0.07
Ce	26.0	0.26	22.6	0.09	28.7	0.46	12.2	0.10
Pr	4.28	0.04	3.75	0.05	4.76	0.10	1.91	0.01
Nd	21.7	0.41	19.1	0.34	24.0	0.31	9.38	0.14
Sm	7.49	0.08	6.67	0.06	8.22	0.12	3.26	0.04
Eu	1.80	0.02	1.79	0.04	1.98	0.02	1.07	0.01
Gd	9.04	0.20	8.12	0.06	9.86	0.11	4.14	0.03
Tb	1.53	0.03	1.36	0.03	1.68	0.04	0.71	0.01
Dy	9.60	0.23	8.55	0.16	10.5	0.14	4.51	0.10
Ho	1.96	0.03	1.76	0.04	2.13	0.02	0.96	0.01
Er	5.04	0.10	4.51	0.06	5.56	0.08	2.51	0.06
Tm	0.72	0.01	0.64	0.01	0.77	0.02	0.36	0.01
Yb	4.41	0.05	3.94	0.06	4.78	0.10	2.31	0.03
Lu	0.63	0.01	0.56	0.01	0.67	0.01	0.32	0.01
Hf	5.67	0.10	5.01	0.06	6.12	0.06	2.36	0.05
Ta	0.44	0.01	0.38	0.01	0.47	0.01	0.27	0.00
Pb	0.49	0.01	0.48	0.01	0.56	0.01	0.51	0.01
Th	0.98	0.03	0.85	0.01	1.08	0.02	0.62	0.01
U	0.28	0.00	0.23	0.01	0.29	0.00	0.17	0.01

Table S5.
Summary of experimental conditions and results.

Run No.	P (GPa)	T (°C)	Duration (h)	fO_2 buffer	Phases	Modal proportions (%)
C19	1.3	1230	48	Pt+graphite	cpx, melt	17: 83
C20	1.3	1200	48	Pt+graphite	cpx, melt	26: 74
C21	1.3	1170	48	Pt+graphite	cpx, grt, ilm, melt	35: 20: 1: 44
M5	1.0	1230	48	Pt+graphite	ch, melt	<1: 99
M6	1.0	1200	48	Pt+graphite	ch, cpx, melt	<1: 8: 91
C17	1.0	1170	48	Pt+graphite	ch, cpx, melt	<1: 21: 79
N4	0.7	1170	48	Pt+graphite	ch, melt	<1: 99
C15	0.7	1140	48	Pt+graphite	ch, cpx, pl, melt	1: 25: 1: 73
C16	0.7	1110	48	Pt+graphite	cpx, pl, ilm, melt	38: 11: 4: 47
N3	0.4	1140	48	Pt+graphite	ch, melt	<1: 99
C13	0.4	1120	48	Pt+graphite	ch, ol, pl, melt	2: 4: 2: 92
C14	0.4	1100	48	Pt+graphite	ol, cpx, pl, ilm, melt	9: 1: 9: 1: 80

Pl-plagioclase, cpx-clinopyroxene, ilm-ilmenite, ol-olivine, ch-chromite, grt-garnet.

Data S1. (.xlsx file)

Pb isotopes of Zr-bearing minerals in Chang'e 6 very low-Ti basaltic clasts.

Data S2. (.xlsx file)

EPMA data of pyroxene and plagioclase in Chang'e 6 low-Ti and very low-Ti basaltic clasts.

Data S3. (.xlsx file)

EPMA data of phases produced in the simulation experiments.

Data S4. (.xlsx file)

Global TiO_2 abundance (obtained by remote sensing survey) and model ages of mare basalts (obtained by crater counting method).

Data S5. (.xlsx file)

Non-modal mantle melting modeling of Zr/Nb-Ta/Nd ratios in mare basalts.

REFERENCES AND NOTES

1. J. F. Snape, A. A. Nemchin, M. J. Whitehouse, R. E. Merle, T. Hopkinson, M. Anand, The timing of basaltic volcanism at the Apollo landing sites. *Geochim. Cosmochim. Acta* **266**, 29–53 (2019).

2. L. Nyquist, C.-Y. Shih, The isotopic record of lunar volcanism. *Geochim. Cosmochim. Acta* **56**, 2213–2234 (1992).

3. K. H. Joy, T. Arai, Lunar meteorites: New insights into the geological history of the Moon. *Astron. Geophys.* **54**, 4.28–24.32 (2013).

4. H. Hiesinger, J. Head, U. Wolf, R. Jaumann, G. Neukum, Ages and stratigraphy of lunar mare basalts: A synthesis. *Geol. Soc. Am. Spec. Pap.* **477**, 1–51 (2011).

5. T. Morota, J. Haruyama, M. Ohtake, T. Matsunaga, C. Honda, Y. Yokota, J. Kimura, Y. Ogawa, N. Hirata, H. Demura, Timing and characteristics of the latest mare eruption on the Moon. *Earth Planet. Sci. Lett.* **302**, 255–266 (2011).

6. J. H. Pasckert, H. Hiesinger, C. H. van der Bogert, Small-scale lunar farside volcanism. *Icarus* **257**, 336–354 (2015).

7. Q.-L. Li, Q. Zhou, Y. Liu, Z. Xiao, Y. Lin, J.-H. Li, H.-X. Ma, G.-Q. Tang, S. Guo, X. Tang, Two-billion-year-old volcanism on the Moon from Chang'e-5 basalts. *Nature* **600**, 54–58 (2021).

8. X. Che, A. Nemchin, D. Liu, T. Long, C. Wang, M. D. Norman, K. H. Joy, R. Tartese, J. Head, B. Jolliff, J. F. Snape, C. R. Neal, M. J. Whitehouse, C. Crow, G. Benedix, F. Jourdan, Z. Yang, C. Yang, J. Liu, S. Xie, Z. Bao, R. Fan, D. Li, Z. Li, S. G. Webb, Age and composition of young basalts on the Moon, measured from samples returned by Chang'e-5. *Science* **374**, 887–890 (2021).

9. L. E. Borg, C. K. Shearer, Y. Asmerom, J. J. Papike, Prolonged KREEP magmatism on the Moon indicated by the youngest dated lunar igneous rock. *Nature* **432**, 209–211 (2004).

10. H.-C. Tian, H. Wang, Y. Chen, W. Yang, Q. Zhou, C. Zhang, H.-L. Lin, C. Huang, S.-T. Wu, L.-H. Jia, L. Xu, D. Zhang, X. G. Li, R. Chang, Y. H. Yang, L. W. Xie, D. P. Zhang, G. L. Zhang, S. H. Yang, F. Y. Wu, Non-KREEP origin for Chang'e-5 basalts in the Procellarum KREEP Terrane. *Nature* **600**, 59–63 (2021).

11. S. M. Elardo, C. K. Shearer, K. E. Vander Kaaden, F. M. McCubbin, A. S. Bell, Petrogenesis of primitive and evolved basalts in a cooling Moon: Experimental constraints from the youngest known lunar magmas. *Earth Planet. Sci. Lett.* **422**, 126–137 (2015).

12. L. Hallis, M. Anand, S. Strekopytov, Trace-element modelling of mare basalt parental melts: Implications for a heterogeneous lunar mantle. *Geochim. Cosmochim. Acta* **134**, 289–316 (2014).

13. G. A. Snyder, L. A. Taylor, C. R. Neal, A chemical model for generating the sources of mare basalts: Combined equilibrium and fractional crystallization of the lunar magmasphere. *Geochim. Cosmochim. Acta* **56**, 3809–3823 (1992).

14. S. Elardo, K. Cone, S. Williams, R. Palin, A shallow mantle source for the Chang'e 5 basalts indicates prolonged indirect heating of the upper mantle by KREEP. *LPI Contributions* **2806**, 2624 (2023).

15. C. Haupt, C. Renggli, M. Klaver, E. Steenstra, J. Berndt, A. Rohrbach, S. Klemme, Experimental and petrological investigations into the origin of the lunar Chang'e 5 basalts. *Icarus* **402**, 115625 (2023).

16. C. Wang, Y.-G. Xu, L. Zhang, Z. Chen, X. Xia, M. Lin, F. Guo, A shallow (< 100 km) ilmenite-bearing pyroxenitic source for young lunar volcanism. *Earth Planet. Sci. Lett.* **639**, 118770 (2024).

17. Y. Qian, J. Head, J. Michalski, X. Wang, C. H. van der Bogert, H. Hiesinger, L. Sun, W. Yang, L. Xiao, X. Li, Long-lasting farside volcanism in the Apollo basin: Chang'e-6 landing site. *Earth Planet. Sci. Lett.* **637**, 118737 (2024).

18. D. Lawrence, W. Feldman, B. Barraclough, A. Binder, R. Elphic, S. Maurice, D. Thomsen, Global elemental maps of the Moon: The Lunar Prospector gamma-ray spectrometer. *Science* **281**, 1484–1489 (1998).

19. M. T. Zuber, D. E. Smith, F. G. Lemoine, G. A. Neumann, The shape and internal structure of the Moon from the Clementine mission. *Science* **266**, 1839–1843 (1994).

20. Z. Cui, Q. Yang, Y.-Q. Zhang, C. Wang, H. Xian, Z. Chen, Z. Xiao, Y. Qian, J. W. Head III, C. R. Neal, A sample of the Moon's far side retrieved by Chang'e-6 contains 2.83-billion-year-old basalt. *Science* **386**, 1395–1399 (2024).

21. C. Li, H. Hu, M.-F. Yang, J. Liu, Q. Zhou, X. Ren, B. Liu, D. Liu, X. Zeng, W. Zuo, Nature of the lunar far-side samples returned by the Chang'E-6 mission. *Natl. Sci. Rev.* **11**, nwae328 (2024).

22. Q. W. L. Zhang, M.-H. Yang, Q.-L. Li, Y. Liu, Z.-Y. Yue, Q. Zhou, L.-Y. Chen, H.-X. Ma, S.-H. Yang, X. Tang, Lunar farside volcanism 2.8 billion years ago from Chang'e-6 basalts. *Nature* **643**, 356–360 (2025).

23. X. Che, T. Long, A. Nemchin, S. Xie, L. Qiao, Z. Li, Y. Ban, R. Fan, C. Yang, D. Liu, Isotopic and compositional constraints on the source of basalt collected from the lunar farside. *Science* **387**, 1306–1310 (2025).

24. C. R. Neal, L. A. Taylor, Petrogenesis of mare basalts: A record of lunar volcanism. *Geochim. Cosmochim. Acta* **56**, 2177–2211 (1992).

25. L. V. Danyushevsky, P. Plechov, Petrolog3: Integrated software for modeling crystallization processes. *Geochem. Geophys. Geosyst.* **12**, Q07021 (2011).

26. B. Su, J. Yuan, Y. Chen, W. Yang, R. N. Mitchell, H. Hui, H. Wang, H. Tian, X.-H. Li, F.-Y. Wu, Fusible mantle cumulates trigger young mare volcanism on the Cooling Moon. *Sci. Adv.* **8**, eabn2103 (2022).

27. S. Klemme, D. Günther, K. Hametner, S. Prowatke, T. Zack, The partitioning of trace elements between ilmenite, ulvospinel, armalcolite and silicate melts with implications for the early differentiation of the moon. *Chem. Geol.* **234**, 251–263 (2006).

28. N. Dygert, Y. Liang, C. Sun, P. Hess, An experimental study of trace element partitioning between augite and Fe-rich basalts. *Geochim. Cosmochim. Acta* **132**, 170–186 (2014).

29. J. A. van Orman, T. L. Grove, Origin of lunar high-titanium ultramafic glasses: Constraints from phase relations and dissolution kinetics of clinopyroxene-ilmenite cumulates. *Meteorit. Planet. Sci.* **35**, 783–794 (2000).

30. C. Münker, A high field strength element perspective on early lunar differentiation. *Geochim. Cosmochim. Acta* **74**, 7340–7361 (2010).

31. K. B. Prissel, M. J. Krawczynski, N. X. Nie, N. Dauphas, S. M. Aarons, A. W. Heard, M. Y. Hu, E. E. Alp, J. Zhao, Fractionation of iron and titanium isotopes by ilmenite and the isotopic compositions of lunar magma ocean cumulates. *Geochim. Cosmochim. Acta* **372**, 154–170 (2024).

32. B. Charlier, T. L. Grove, O. Namur, F. Holtz, Crystallization of the lunar magma ocean and the primordial mantle-crust differentiation of the Moon. *Geochim. Cosmochim. Acta* **234**, 50–69 (2018).

33. J. Rapp, D. Draper, Fractional crystallization of the lunar magma ocean: Updating the dominant paradigm. *Meteorit. Planet. Sci.* **53**, 1432–1455 (2018).

34. Y. Lin, E. J. Tronche, E. S. Steenstra, W. van Westrenen, Experimental constraints on the solidification of a nominally dry lunar magma ocean. *Earth Planet. Sci. Lett.* **471**, 104–116 (2017).

35. C. P. Haupt, C. J. Renggli, A. Rohrbach, J. Berndt, S. Schwinger, M. Maurice, M. Schulze, D. Breuer, S. Klemme, Trace element partitioning in the lunar magma ocean: An experimental study. *Contrib. Mineral. Petrol.* **179**, 45 (2024).

36. J. Longhi, Experimental petrology and petrogenesis of mare volcanics. *Geochim. Cosmochim. Acta* **56**, 2235–2251 (1992).

37. T. L. Grove, M. J. Krawczynski, Lunar mare volcanism: Where did the magmas come from? *Elements* **5**, 29–34 (2009).

38. H. Xiang, J. A. Connolly, GeoPS: An interactive visual computing tool for thermodynamic modelling of phase equilibria. *J. Metam. Geol.* **40**, 243–255 (2022).

39. D. A. Neave, K. D. Putirka, A new clinopyroxene-liquid barometer, and implications for magma storage pressures under Icelandic rift zones. *Am. Mineral.* **102**, 777–794 (2017).

40. K. D. Putirka, Thermometers and barometers for volcanic systems. *Rev. Mineral. Geochem.* **69**, 61–120 (2008).

41. L. T. Elkins-Tanton, S. Burgess, Q.-Z. Yin, The lunar magma ocean: Reconciling the solidification process with lunar petrology and geochronology. *Earth Planet. Sci. Lett.* **304**, 326–336 (2011).

42. Y. Zhao, J. De Vries, A. van den Berg, M. Jacobs, W. van Westrenen, The participation of ilmenite-bearing cumulates in lunar mantle overturn. *Earth Planet. Sci. Lett.* **511**, 1–11 (2019).

43. A. Mallik, T. Ejaz, S. Shcheka, G. Garapic, A petrologic study on the effect of mantle overturn: Implications for evolution of the lunar interior. *Geochim. Cosmochim. Acta* **250**, 238–250 (2019).

44. M. Xu, Z. Jing, J. A. Van Orman, T. Yu, Y. Wang, Experimental evidence supporting an overturned iron-titanium-rich melt layer in the deep lunar interior. *Geophys. Res. Lett.* **49**, e2022GL099066 (2022).

45. P. C. Hess, E. Parmentier, A model for the thermal and chemical evolution of the Moon's interior: Implications for the onset of mare volcanism. *Earth Planet. Sci. Lett.* **134**, 501–514 (1995).

46. N. Zhang, M. Ding, M.-H. Zhu, H. Li, H. Li, Z. Yue, Lunar compositional asymmetry explained by mantle overturn following the South Pole–Aitken impact. *Nat. Geosci.* **15**, 37–41 (2022).

47. M. Klaver, S. Klemme, X.-N. Liu, R. C. Hin, C. D. Coath, M. Anand, C. J. Lissenberg, J. Berndt, T. Elliott, Titanium-rich basaltic melts on the Moon modulated by reactive flow processes. *Nat. Geosci.* **17**, 118–123 (2024).

48. B. Su, Y. Chen, Z. Wang, D. Zhang, H. Chen, S. Gou, Z. Yue, Y. Liu, J. Yuan, G. Tang, South Pole–Aitken massive impact 4.25 billion years ago revealed by Chang'e-6 samples. *Natl. Sci. Rev.* **12**, nwaf103 (2025).

49. K. Joy, N. Wang, J. Snape, A. Goodwin, J. Pernet-Fisher, M. J. Whitehouse, Y. Liu, Y. Lin, J. Darling, P. Tar, Evidence of a 4.33 billion year age for the Moon's South Pole–Aitken basin. *Nat. Astron.* **9**, 55–65 (2025).

50. W. M. Vaughan, J. W. Head, Impact melt differentiation in the South Pole-Aitken basin: Some observations and speculations. *Planet. Space Sci.* **91**, 101–106 (2014).

51. D. M. Hurwitz, D. A. Kring, Differentiation of the South Pole–Aitken basin impact melt sheet: Implications for lunar exploration. *J. Geophys. Res. Planets* **119**, 1110–1133 (2014).

52. D. Moriarty Iii, R. Watkins, S. Valencia, J. Kendall, A. Evans, N. Dygert, N. Petro, Evidence for a stratified upper mantle preserved within the South Pole-Aitken basin. *J. Geophys. Res. Planets* **126**, e2020JE006589 (2021).

53. F. Nimmo, T. Kleine, A. Morbidelli, Tidally driven remelting around 4.35 billion years ago indicates the Moon is old. *Nature* **636**, 598–602 (2024).

54. R. Ziethe, K. Seiferlin, H. Hiesinger, Duration and extent of lunar volcanism: Comparison of 3D convection models to mare basalt ages. *Planet. Space Sci.* **57**, 784–796 (2009).

55. S. Cai, K. Qi, S. Yang, J. Fang, P. Shi, Z. Shen, M. Zhang, H. Qin, C. Zhang, X. Li, A reinforced lunar dynamo recorded by Chang'e-6 farside basalt. *Nature* **643**, 361–365 (2024).

56. S. Cai, H. Qin, H. Wang, C. Deng, S. Yang, Y. Xu, C. Zhang, X. Tang, L. Gu, X. Li, Persistent but weak magnetic field at the Moon's midstage revealed by Chang'e-5 basalt. *Sci. Adv.* **11**, eadp3333 (2025).

57. L. Wilson, J. W. Head, Generation, ascent and eruption of magma on the Moon: New insights into source depths, magma supply, intrusions and effusive/explosive eruptions (Part 1: Theory). *Icarus* **283**, 146–175 (2017).

58. J. W. Head, L. Wilson, Generation, ascent and eruption of magma on the Moon: New insights into source depths, magma supply, intrusions and effusive/explosive eruptions (Part 2: Predicted emplacement processes and observations). *Icarus* **283**, 176–223 (2017).

59. Q. Deng, Z. Xiao, Y. Wu, P. Ma, W. Cao, Y. Wang, Y. Ma, F. Luo, F. Li, Magmatic dikes in the Chang'e-6 sampling area. *Earth Planet. Sci. Lett.* **660**, 119350 (2025).

60. M. Daines, D. L. Kohlstedt, The transition from porous to channelized flow due to melt/rock reaction during melt migration. *Geophys. Res. Lett.* **21**, 145–148 (1994).

61. P. G. Lucey, D. T. Blewett, B. L. Jolliff, Lunar iron and titanium abundance algorithms based on final processing of Clementine ultraviolet-visible images. *J. Geophys. Res. Planets* **105**, 20297–20305 (2000).

62. J. Whitten, J. W. Head, M. Staid, C. M. Pieters, J. Mustard, R. Clark, J. Nettles, R. L. Klima, L. Taylor, Lunar mare deposits associated with the Orientale impact basin: New insights into mineralogy, history, mode of emplacement, and relation to Orientale Basin evolution from Moon Mineralogy Mapper (M3) data from Chandrayaan-1. *J. Geophys. Res. Planets* **116**, 1–33 (2011).

63. J. T. Wasson, P. H. Warren, Contribution of the mantle to the lunar asymmetry. *Icarus* **44**, 752–771 (1980).

64. Y. Srivastava, A. Basu Sarbadhikari, J. M. Day, A. Yamaguchi, A. Takenouchi, A changing thermal regime revealed from shallow to deep basalt source melting in the Moon. *Nat. Commun.* **13**, 7594 (2022).

65. K. P. Jochum, M. Willbold, I. Raczek, B. Stoll, K. Herwig, Chemical characterisation of the USGS reference glasses GSA-1G, GSC-1G, GSD-1G, GSE-1G, BCR-2G, BHVO-2G and BIR-1G Using EPMA, ID-TIMS, ID-ICP-MS and LA-ICP-MS. *Geostand. Geoanal. Res.* **29**, 285–302 (2005).

66. T. Reischmann, Precise U/Pb age determination with baddeleyite (Zr02), a case study from the Phalaborwa igneous complex, South Africa. *S. Afr. J. Geol.* **98**, 1–4 (1995).

67. E. Catanzaro, T. J. Murphy, E. Garner, W.-R. Shields, Absolute isotopic abundance ratio and atomic weight of terrestrial rubidium. *J. Res. Natl. Stand. A. Phys. Chem.* **73A**, 511–516 (1969).

68. J. Woodhead, S. Swearer, J. Hergt, R. Maas, In situ Sr-isotope analysis of carbonates by LA-MC-ICP-MS: Interference corrections, high spatial resolution and an example from otolith studies. *J. Anal. At. Spectrom* **20**, 22–27 (2005).

69. F. C. Ramos, J. A. Wolff, D. L. Tollstrup, Measuring 87Sr/86Sr variations in minerals and groundmass from basalts using LA-MC-ICPMS. *Chem. Geol.* **211**, 135–158 (2004).

70. L. Zhang, Z. Y. Ren, Y. D. Wu, N. Li, Strontium isotope measurement of basaltic glasses by laser ablation multiple collector inductively coupled plasma mass spectrometry based on a linear relationship between analytical bias and Rb/Sr ratios. *Rapid Commun. Mass Spectrom.* **32**, 105–112 (2018).

71. J.-I. Kimura, T. Takahashi, Q. Chang, A new analytical bias correction for in situ Sr isotope analysis of plagioclase crystals using laser-ablation multiple-collector inductively coupled plasma mass spectrometry. *J. Anal. At. Spectrom* **28**, 945–957 (2013).

72. M. G. Nicholis, M. J. Rutherford, Graphite oxidation in the Apollo 17 orange glass magma: Implications for the generation of a lunar volcanic gas phase. *Geochim. Cosmochim. Acta* **73**, 5905–5917 (2009).

73. M. Sato, N. L. Hickling, J. E. McLane, in *Proceedings of the Lunar Science Conference* (Pergamon Press, 1973), vol. 4, pp. 1061.

74. Z. Jia, J. Chen, J. Kong, L. Qiao, X. Fu, Z. Ling, Geologic context of Chang'e-6 candidate landing regions and potential non-mare materials in the returned samples. *Icarus* **416**, 116107 (2024).

75. C. Li, H. Hu, M.-F. Yang, Z.-Y. Pei, Q. Zhou, X. Ren, B. Liu, D. Liu, X. Zeng, G. Zhang, Characteristics of the lunar samples returned by the Chang'E-5 mission. *Natl. Sci. Rev.* **9**, nwab188 (2022).

76. K. Zong, Z. Wang, J. Li, Q. He, Y. Li, H. Becker, W. Zhang, Z. Hu, T. He, K. Cao, Bulk compositions of the Chang'E-5 lunar soil: Insights into chemical homogeneity, exotic addition, and origin of landing site basalts. *Geochim. Cosmochim. Acta* **335**, 284–296 (2022).

77. K. A. Cone, ApolloBasalt DB_V2, version 1.0, Interdisciplinary Earth Data Alliance (IEDA) (2021).

78. C. R. Neal, Interior of the Moon: The presence of garnet in the primitive deep lunar mantle. *J. Geophys. Res. Planets* **106**, 27865–27885 (2001).

79. Q. He, Y. Li, I. Baziotis, Y. Qian, L. Xiao, Z. Wang, W. Zhang, B. Luo, C. R. Neal, J. M. Day, Detailed petrogenesis of the unsampled Oceanus Procellarum: The case of the Chang'e-5 mare basalts. *Icarus* **383**, 115082 (2022).

80. K. L. Robinson, A. H. Treiman, K. H. Joy, Basaltic fragments in lunar feldspathic meteorites: Connecting sample analyses to orbital remote sensing. *Meteorit. Planet. Sci.* **47**, 387–399 (2012).

81. P. Beattie, Olivine-melt and orthopyroxene-melt equilibria. *Contrib. Mineral. Petrol.* **115**, 103–111 (1993).

82. L. V. Danyushevsky, The effect of small amounts of H_2O on crystallisation of mid-ocean ridge and backarc basin magmas. *J. Volcanol. Geotherm. Res.* **110**, 265–280 (2001).

83. R. L. Nielsen, EQUIL: A program for the modeling of low-pressure differentiation processes in natural mafic magma bodies. *Comput. Geosci.* **11**, 531–546 (1985).

84. N. J. Potts, R. Tartèse, M. Anand, W. van Westrenen, A. A. Griffiths, T. J. Barrett, I. A. Franchi, Characterization of mesostasis regions in lunar basalts: Understanding late-stage melt evolution and its influence on apatite formation. *Meteorit. Planet. Sci.* **51**, 1555–1575 (2016).

85. X. Wang, T. Hou, M. Wang, C. Zhang, Z. Zhang, R. Pan, F. Marxer, H. Zhang, A new clinopyroxene thermobarometer for mafic to intermediate magmatic systems. *Eur. J. Mineral.* **33**, 621–637 (2021).

86. T. Holland, R. Powell, An improved and extended internally consistent thermodynamic dataset for phases of petrological interest, involving a new equation of state for solids. *J. Metam. Geol.* **29**, 333–383 (2011).

87. E. S. Jennings, T. J. Holland, A simple thermodynamic model for melting of peridotite in the system NCFMASOCr. *J. Petrol.* **56**, 869–892 (2015).

88. D. Walker, R. Kirkpatrick, J. Longhi, J. Hays, Crystallization history of lunar picritic basalt sample 12002: Phase-equilibria and cooling-rate studies. *Geol. Soc. Am. Bull.* **87**, 646–656 (1976).

89. N. Zhang, E. Parmentier, Y. Liang, A 3-D numerical study of the thermal evolution of the Moon after cumulate mantle overturn: The importance of rheology and core solidification. *J. Geophys. Res. Planets* **118**, 1789–1804 (2013).

90. M. Laneuville, M. Wieczorek, D. Breuer, N. Tosi, Asymmetric thermal evolution of the Moon. *J. Geophys. Res. Planets* **118**, 1435–1452 (2013).

91. G. V. Depine, C. L. Andronicos, J. Phipps-Morgan, Near-isothermal conditions in the middle and lower crust induced by melt migration. *Nature* **452**, 80–83 (2008).

92. R. Nielsen, M. Drake, in *Mare Crisium: The View From Luna* (Pergamon Press, 1978), vol. 24, pp. 419–428.

93. D. Vaniman, J. Papike, Ferrobasalts from Mare Crisium: Luna 24. *Geophys. Res. Lett.* **4**, 497–500 (1977).

94. J. Rhodes, D. Blanchard, in *Lunar and Planetary Science Conference, 11th, Houston, TX, March 17-21, 1980, Proceedings. Volume 1 (A82–22251 09–91)* (Pergamon Press, 1980), vol. 11, pp. 49–66.

95. G. A. Snyder, C. R. Neal, L. A. Taylor, A. N. Halliday, Anatexis of lunar cumulate mantle in time and space: Clues from trace-element, strontium, and neodymium isotopic chemistry of parental Apollo 12 basalts. *Geochim. Cosmochim. Acta* **61**, 2731–2747 (1997).

96. W. F. McDonough, S.-S. Sun, The composition of the Earth. *Chem. Geol.* **120**, 223–253 (1995).

97. L. T. Elkins-Tanton, B. H. Hager, T. L. Grove, Magmatic effects of the lunar late heavy bombardment. *Earth Planet. Sci. Lett.* **222**, 17–27 (2004).

98. S. M. Elardo, C. K. Shearer Jr, A. L. Fagan, L. E. Borg, A. M. Gaffney, P. V. Burger, C. R. Neal, V. A. Fernandes, F. M. McCubbin, The origin of young mare basalts inferred from lunar meteorites Northwest Africa 4734, 032, and LaPaz Icefield 02205. *Meteorit. Planet. Sci.* **49**, 261–291 (2014).

99. L. E. Borg, A. M. Gaffney, C. K. Shearer, D. J. DePaolo, I. D. Hutcheon, T. L. Owens, E. Ramon, G. Brennecka, Mechanisms for incompatible-element enrichment on the Moon deduced from the lunar basaltic meteorite Northwest Africa 032. *Geochim. Cosmochim. Acta* **73**, 3963–3980 (2009).

100. F. Hodges, I. Kushiro, Liquidus phase relations of Apollo 15 mare basalt 15016. *Carnegie Institute Washington Yearbook* **7**, 646–647 (1972).

101. S. Kesson, in *Lunar Science Conference Proceedings, 6th, Houston, Texas, March 17-21, 1975, Volume 1 (A78–46603 21–91)* (Pergamon Press, 1975), vol. 6, pp. 921–944.

102. N. Vlaar, P. Van Keken, A. Van den Berg, Cooling of the Earth in the Archaean: Consequences of pressure-release melting in a hotter mantle. *Earth Planet. Sci. Lett.* **121**, 1–18 (1994).
